# Recent advances in developing active targeting and multi-functional drug delivery systems via bioorthogonal chemistry

**DOI:** 10.1038/s41392-022-01250-1

**Published:** 2022-12-02

**Authors:** Wenzhe Yi, Ping Xiao, Xiaochen Liu, Zitong Zhao, Xiangshi Sun, Jue Wang, Lei Zhou, Guanru Wang, Haiqiang Cao, Dangge Wang, Yaping Li

**Affiliations:** 1 State Key Laboratory of Drug Research & Center of Pharmaceutics, Shanghai Institute of Materia Medica, Chinese Academy of Sciences, Shanghai, 201203 China; 2 Yantai Key Laboratory of Nanomedicine & Advanced Preparations, Yantai Institute of Materia Medica, Yantai, 264000 China; 3 Shandong Laboratory of Yantai Drug Discovery, Bohai Rim Advanced Research Institute for Drug Discovery, Yantai, 264000 China

**Keywords:** Metabolic engineering, Biomaterials

## Abstract

Bioorthogonal chemistry reactions occur in physiological conditions without interfering with normal physiological processes. Through metabolic engineering, bioorthogonal groups can be tagged onto cell membranes, which selectively attach to cargos with paired groups via bioorthogonal reactions. Due to its simplicity, high efficiency, and specificity, bioorthogonal chemistry has demonstrated great application potential in drug delivery. On the one hand, bioorthogonal reactions improve therapeutic agent delivery to target sites, overcoming off-target distribution. On the other hand, nanoparticles and biomolecules can be linked to cell membranes by bioorthogonal reactions, providing approaches to developing multi-functional drug delivery systems (DDSs). In this review, we first describe the principle of labeling cells or pathogenic microorganisms with bioorthogonal groups. We then highlight recent breakthroughs in developing active targeting DDSs to tumors, immune systems, or bacteria by bioorthogonal chemistry, as well as applications of bioorthogonal chemistry in developing functional bio-inspired DDSs (biomimetic DDSs, cell-based DDSs, bacteria-based and phage-based DDSs) and hydrogels. Finally, we discuss the difficulties and prospective direction of bioorthogonal chemistry in drug delivery. We expect this review will help us understand the latest advances in the development of active targeting and multi-functional DDSs using bioorthogonal chemistry and inspire innovative applications of bioorthogonal chemistry in developing smart DDSs for disease treatment.

## Introduction

Many therapeutic candidates have had difficulty translating to clinical applications due to high drug accumulation in non-target organs and low enrichment in target organs.^1^ Drug delivery systems (DSSs) have enabled the development of many therapeutic agents that promote patient compliance and improve patient health by enhancing their delivery to the target sites and minimizing off-target accumulation.^2,3^ Current DDSs primarily consist of synthetic organic material systems (e.g., liposomes, micelles, and polymeric nanoparticles^4–6^), inorganic material systems (e.g., mesoporous silica nanoparticles and metal-organic frameworks^7–9^), and bio-inspired DDSs (e.g., viral-based, biomimetic and cell-based DDSs^10–15^). As treatment methods evolve from small molecule chemicals to nucleic acids, peptides, antibodies, and living cells, DDSs are facing numerous obstacles.^16^ For example, nanomedicines can transport therapeutic drugs to the target site through enhanced permeability and retention (EPR) effects,^17–20^ but they still accumulate in large quantities around blood vessels, preventing drug penetration into diseased tissue.^21–23^ Although several studies have shown that modifying specific ligands on the surface of nanomedicines, such as folic acid, peptides, and antibodies, can improve targeting ability,^24–26^ the targeting effectiveness is mostly dependent on the different expression of receptors on target cells and other cells.^27–30^ In terms of microenvironmental response strategies, DDSs can respond to specific physicochemical properties or overexpressed enzymes of the microenvironment, but the minor differences in acidity or enzyme activity between normal and diseased tissues make it difficult to develop nanomaterials sensitive to such modest changes.^31–34^ As for bio-inspired DDSs, the inherent defects of the cell limits their development. For example, while chimeric antigen receptor T cells (CAR-T) can destroy tumor cells, they cannot penetrate deep into tumor tissue, limiting their ability.^35,36^ Although genetic engineering technologies are developed to endow cells with new functions, they are time-consuming, costly, and have a poor success rate, leading to a long production cycle.^37–42^ Therefore, new strategies are needed to overcome the challenges of DDSs. As an emerging chemical modification technique, bioorthogonal chemistry has shown enormous potential in developing DDSs.

Bioorthogonal reactions happen in living cells without interfering with the normal biochemical functions of the organism.^43–45^ The first bioorthogonal reaction is a Staudinger ligation that occurs between N_3_ and a triarylphosphine group.^45^ However, due to its poor reactivity, this reaction has not been widely exploited in biological research.^46–48^ In 2002, Sharpless et al. proposed Copper(I)-catalyzed azide-alkyne 1,3-dipolar cycloaddition (CuAAC) which exhibits a much higher reaction rate (10–100 M^−1^ s^−1^ with 1 mol% Cu(I)) than Staudinger reaction (0.008 M^−1^ s^−1^) and better selectivity.^49,50^ However, CuAAC cannot be used in living cells because of copper(I)-induced cytotoxicity. Although various methods have been employed to reduce toxicity, they do not address the core cause of the problem.^51–53^ To overcome the copper cytotoxicity of CuAAC, Bertozzi and co-workers employed electron-deficient deformed alkynes to construct a strain-promoted azide-alkyne cycloaddition (SPAAC), which showed a great reactivity(1–60 M^−1^ s^−1^) with no copper cytotoxicity.^54–56^ Following that, Fox and his team created the inverse electron demand Diels-Alder (iEDDA) process (1–10^6^ M^−1^ s^−1^), which is based on the cycloaddition reaction of tetrazine (Tz) and trans-cyclooctene (TCO) derivatives and has a faster reaction rate than SPAAC.^57,58^ Considering that numerous chemical reactants with high specificity to each other in a test tube will lose selectivity due to the interference of various biologically active components, these copper-free bioorthogonal reactions are more suitable for biomedical research because of their nontoxicity, efficiency, and high selectivity in biological systems. Even under adverse physiological conditions such as the acidic and hypoxic environment, these reactions are rapid and effective, with minimal cell damage.^59–63^

In general, applying copper-free bioorthogonal chemistry in living organisms involves two steps. First, bioorthogonal groups (such as N_3_) must be inserted into target biomolecules on the cell membrane. Metabolic engineering, an emerging cell engineering strategy, has been used in recent years to conjugate functional groups into biomolecules such as proteins, glycans, and phospholipids on the cell membrane by utilizing the cells’ inherent metabolic pathways.^45,59,64–68^ Bioorthogonal groups can be labeled on the cell membrane in vitro or in vivo by modifying them with amino acids, monosaccharides, and choline because cells must utilize these metabolic precursors during innate biosynthetic pathways. Second, nanoparticles or antibodies with pairing groups (such as dibenzyl cyclooctyne (DBCO)) conjugate to bioorthogonal groups (such as N_3_) labeled cells via bioorthogonal reactions.^69,70^ Based on these processes, bioorthogonal chemistry has been employed to improve the targeting delivery of therapeutics and develop DDSs.^71,72^ On the one hand, bioorthogonal groups can be presented on the cell membrane in great density by metabolic engineering. Therapeutics with paired groups can target cells or tissues with bioorthogonal groups for the treatment of cancer, bacterial infections, and other diseases through bioorthogonal reactions.^73,74^ On the other hand, bioorthogonal reactions are low-cost with high efficiency, allowing the label of cytokines, antibodies, or nanoparticles onto the cell membrane.^12,75^ Cells, bacteria and phages will be endowed with novel functionalities such as selective targeting and immune regulating reactivities, which makes bioorthogonal chemistry a powerful tool to create multi-functional bio-inspired DDSs with higher therapeutic effects.^76^

Given that other reviews have detailed the contribution of bioorthogonal chemistry for cancer imaging, prodrug activation, and cancer therapy but do not detail the application of bioorthogonal chemistry from the developing strategies of DDSs,^77,78^ this review will focus on recent advances in bioorthogonal chemistry (especially SPAAC and iEDDA) in developing DDSs from the perspective of active targeting and multi-functional modifications. We introduced the principle of labeling cells or pathogenic microorganisms with bioorthogonal reactions, including bioorthogonal groups, labeling biomolecules, and labeling strategies in vitro and in vivo. We then highlighted recent breakthroughs in developing active targeting DDSs to tumors, immune systems or bacteria via bioorthogonal chemistry, and its applications in developing functional bio-inspired DDSs. Finally, we discussed the challenges and prospective direction of bioorthogonal chemistry from precision regulation, expansion of toolkit and technology integration for designing novel DDSs. We expect this review will help researchers to overview latest advances in designing active targeting and multi-functional DDSs with bioorthogonal chemistry, and inspire innovative new drugs for disease treatment.

## The principle of bioorthogonal metabolic labeling

**Fig. 1:**
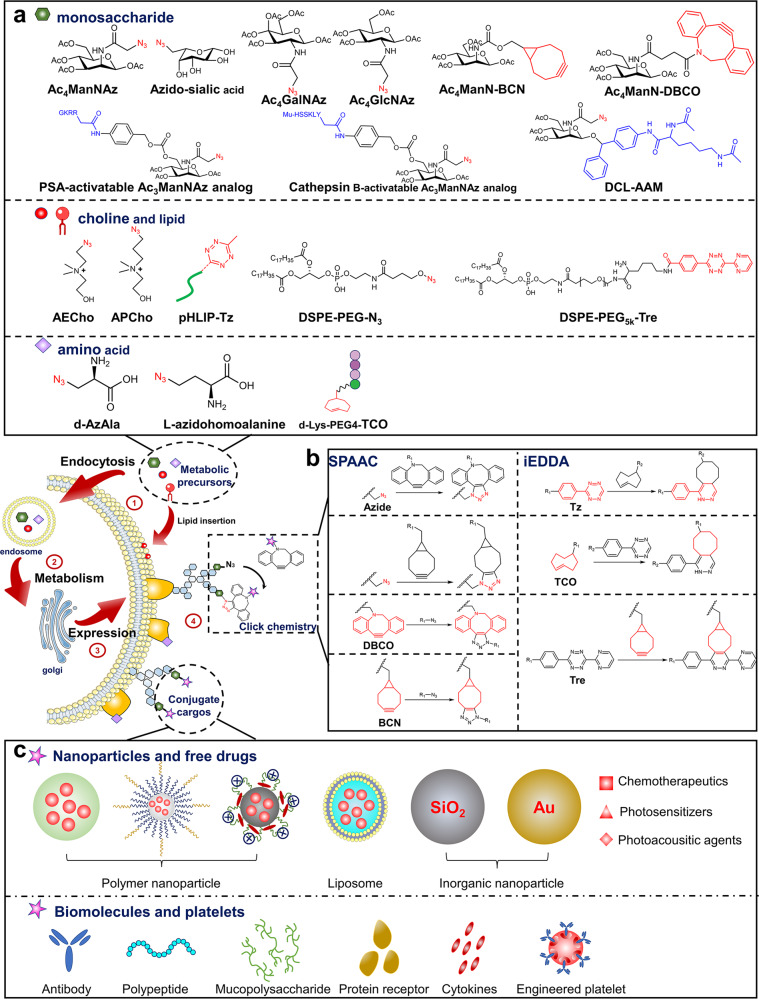
Bioorthogonal metabolic labeling of living cells. First, metabolic precursors are endocytosed by cells. Second, precursors are involved in cellular metabolism. Third, bioorthogonal groups are displayed on the cell surface. Finally, cargos are conjugated to the cell through a click reaction. **a** Metabolic precursors with bioorthogonal groups. Unnatural sugars bind to cell surface glycans through the glucose metabolic pathway. Lipid precursors are embedded in cell membranes through phospholipid synthesis pathways or lipid insertion. Functionalized amino acids bind to membrane proteins through protein synthesis pathways. **b** SPAAC and iEDDA are used for targeted delivery and developing DDSs. **c** Conjugate cargos that bind to cell membranes via bioorthogonal chemistry

Due to the large size of fluorescent labels, cell labeling technologies like fluorescent protein gene coding and dye-antibody conjugation might affect the function of proteins.^71,79–81^ Appling biosynthesis and metabolism systems to introduce bioorthogonal groups into cells or pathogenic bacteria, known as bioorthogonal metabolic labeling, enables living creature labeling. This technology does not affect the structure and function of biological macromolecules, as well as the exchange of substances between cells and the extracellular environment. Furthermore, the high density of bioorthogonal groups provides more binding sites enables for cell membrane engineering and targeted drug delivery. In this section, we will describe chemical groups and target molecules on the cell membrane for bioorthogonal metabolic labeling (Fig. 1). Then, we will go over strategies for in vitro and in vivo cellular bioorthogonal metabolic labeling, providing a reference for delivering metabolic precursors to target cells by nanocarriers or chemical modifications. Finally, we will discuss bioorthogonal metabolic labeling of bacteria and viruses in the hope to generate new ideas for targeted therapy of pathogenic microorganisms.

### Chemical groups for bioorthogonal metabolic labeling

Among various bioorthogonal chemical groups, N_3_ is the most wildly used in cell labeling. Metabolic derivatives like N_3_-modified mannosamine, galactosamine, sialic acid, fucose, glucosamine, and choline can be embedded into cell membrane glycans or phospholipids through cellular metabolism.^73,82–91^ In addition to N_3_, many other bioorthogonal groups have also been inserted into the cell membrane. For example, mannosamine with DBCO can be used to label LS174T colon cancer cells with DBCO.^92^ Alkynyl-modified monosaccharide derivatives can be coupled with N_3_-modified molecules.^93,94^ Furthermore, N-acetylmannosamine (ManNAc), N-acetylgalactosamine (GalNAc), and N-acetylglucosamine (GlcNAc) derivatives with isonitrile groups can be utilized for tumor cell labeling and undergo iEDDA reactions.^95,96^ Given that different bioorthogonal groups have different sizes and physicochemical properties, one of the challenges in bioorthogonal metabolic labeling is identifying and synthesizing the best bioorthogonal groups for cell labeling. The ideal bioorthogonal group should have minimal steric hindrance, good biocompatibility, and mild reaction conditions, as well as a fast reaction rate and high conversion rate. N_3_ and DBCO, TCO, and Tz are the most wildly used bioorthogonal pairing groups in cell labeling which are biocompatible and active in physiological conditions. Although both N_3_ and DBCO can trigger efficient bioorthogonal reactions, N_3_ labeled cells can initiate higher efficiency than DBCO labeled cells.^92^ This could be due to the huge size and steric barrier of DBCO, which makes the ligation reaction more difficult. Additionally, the biocompatibility of bioorthogonal groups is crucial for cell labeling. If bioorthogonal groups degrade quickly, antibodies or nanoparticles will no longer be attached to the cell membrane, resulting in unanticipated adverse side effects. According to research, N_3_ exists on cells longer than other bioorthogonal groups, indicating that N_3_ may now be the best bioorthogonal group.^97^

Furthermore, despite the development of numerous bioorthogonal group-modified metabolic derivatives, there is no clear method for quantifying the absolute number of bioorthogonal groups on the cell surface, making it difficult to compare the labeling efficiency of different metabolic precursors with the same bioorthogonal groups. Moreover, because of the big size of DBCO, not every N_3_ can pair with one DBCO, implying that a large number of N_3_ are not utilized.^98,99^ These challenges hamper our understanding of the differences in bioorthogonal labeling via different metabolic pathways and limit the usage efficiency of bioorthogonal groups on the cell membrane. To more accurately eliminate the number of bioorthogonal groups on the cell membrane, detection reagents of appropriate molecular size or new detection methods must be developed. Finally, the time necessary for bioorthogonal metabolic labeling must be considered. It takes days for cells to be labeled even in vitro, which limits the usage of bioorthogonal metabolic labeling in vivo.^99^ In the future, researchers must create bioorthogonal groups with minimal steric hindrance while preserving stability and biocompatibility. Simultaneously, other cell metabolic pathways could be used to reduce labeling time, resulting in the most efficient and stable bioorthogonal labeling.

### Target sites of bioorthogonal metabolic labeling on the cell membrane

Chemical synthesis links bioorthogonal groups to metabolic precursors such as monosaccharides, amino acids, and choline, which are then introduced into glycans, proteins, and phospholipids via cellular metabolic pathways^81,100^ (Fig. 1a). Bertozzi et al. for example, employed N-azidoacetylmannosamine (Ac_4_ManNAz) to metabolize it into N-azidoacetylneuraminic acid via the sialic acid biosynthetic pathway, allowing N_3_ to be expressed in cytoglycans.^101^ N_3_-modified methionine, leucine, tryptophan, and phenylalanine are incorporated into proteins in *E. coli* or human cells via protein biosynthesis.^102–105^ Choline analogs such as azidoethylcholine (AECho) and azidopropylcholine (APCho) display N_3_ on phospholipids via the intracellular phosphatidylcholine synthesis pathway.^106,107^ Although proteins, phospholipids, and glycans can be utilized as targets for bioorthogonal metabolic labeling, their physiological roles affect the labeling efficacy. Proteins are essential components of living cells. N_3_-modified amino acid derivatives have a similar structure to amino acids and do not affect the structure or function of proteins. However, when paired with DBCO-modified drugs or nanoparticles, the massive steric hindrance may change the spatial structure of the protein, disrupting its normal activity (such as signal transduction and substance transportation). Phospholipids mainly participate in the formation of the cell membrane, thus the addition of N_3_ has little impact on phospholipid function. Furthermore, cell membrane phospholipids have a larger contact surface than proteins, resulting in a greater number of target sites for the bioorthogonal reaction of DBCO and N_3_. In addition to N_3_-modified choline analogs, several synthetic phospholipids have been produced, such as DSPE-PEG_5k_-Tre^108^ and DSPE-PEG-N_3_,^109^ which are implanted in the cell membrane by lipid intercalation. However, bioorthogonal metabolic labeling of phospholipids is mostly used for in vitro cell labeling, because it is difficult to selectively label target cells’ phospholipids in vivo as the component of membrane phospholipids varies little from cell to cell. Glycan labeling is the most wildly used bioorthogonal metabolic labeling technology, which has achieved both in vivo and in vitro cell labeling, including tumor cells,^73,86,89–91^ dendritic cells,^110,111^ and so on. Labeling glycans with bioorthogonal groups has little effect on cell activity. Meanwhile, monosaccharides have more chemical modification sites than amino acids and choline, which can be coupled with high molecular polymers or sensitive linkers. Researchers have developed a variety of bioorthogonal functionalized monosaccharides (also known as unnatural sugars), such as N_3_-modified sialic acid, ManNAc, GalNAc, GlcNAc, fucose, and monosaccharide derivatives with enzyme-responsive groups.^87,98^ Thus, unnatural sugars have received more attention in biomedical research compared to metabolic precursors of phospholipids and proteins.

### Strategies for cell bioorthogonal metabolic labeling in vitro and in vivo

#### In vitro labeling strategy

**Fig. 2:**
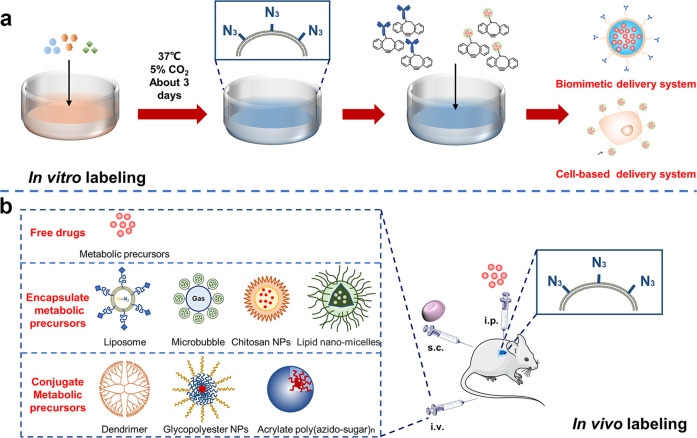
In vitro and in vivo strategies of bioorthogonal metabolic labeling. **a** In vitro bioorthogonal metabolic labeling strategies. Co-incubation of metabolic precursors with cells enables in vitro labeling. **b** In vivo bioorthogonal metabolic labeling strategies. Subcutaneous injection of unnatural sugar-encapsulated gels, intratumoral or intravenous injection of metabolic precursors, and the use of nanocarriers through physical encapsulating or chemical conjugating of metabolic precursors enable bioorthogonal metabolic labeling in vivo

Metabolic labeling of cells in vitro is as simple as adding metabolic precursors to the cell culture media, such as Ac_4_ManNAz, AECho, and APCho (Fig. 2a). These metabolic precursors can be taken up and used by cells instantly, resulting in the labeling of bioorthogonal groups on the cell membrane. Additionally, N_3_ groups can be introduced into the cell membrane via lipid insertion by co-incubating cells with synthetic phospholipids. This in vitro method for bioorthogonal metabolic labeling simplifies the production of bioorthogonal bio-inspired DDSs.

#### In vivo labeling strategy

To realize bioorthogonal metabolic labeling in vivo, metabolic precursors must be delivered to the targeted tissues and absorbed by cells (Fig. 2b). Metabolic precursors can be injected directly into the desired region via local administration. Intratumoral injections, for example, can be utilized for bioorthogonal metabolic labeling in tumors, followed by targeted drug delivery, to create an efficient therapy against cancer. Research has demonstrated that intratumoral injection of Ac_4_ManNAz to A549 tumor-bearing mice could specifically label tumor cells.^112^ Additionally, in the U87 tumor model pre-injected with Ac_4_ManNAz, bicyclononyne (BCN)-modified nanoparticles showed superior tumor targeting capacity than cRGD-modified nanoparticles.^86^ However, because most cancers are difficult to detect, it is difficult to inject metabolic precursors into the tumor site. Another method is systemic delivery, which involves intravenous injection and subcutaneous injection. After the systematical injection of the unnatural sugar organic solution, the unnatural sugar enters the bloodstream, travels to the tumor tissue, and is absorbed by tumor cells. However, this method is limited by low water solubility and pharmacokinetics of unnatural sugars.^88^ To overcome these difficulties, different nanomaterials have been created as delivery vehicles for metabolic precursors to increase circulation stability and tumor targeting. Also, the tumor selectivity of bioorthogonal labeling can be significantly strengthened by modifying metabolic precursors with triggers that can be activated in the tumor microenvironment (TME).

##### Delivering metabolic precursors by nanocarriers

The use of nanocarriers to transport metabolic precursors, notably unnatural sugars, has risen in favor in recent years. On the one hand, nanocarriers can stabilize the pharmacokinetics of unnatural sugars and deliver a substantial number of them at one time. On the other hand, nanocarriers enhance the tumor selectivity of unnatural sugars, reducing the metabolic labeling of normal tissues. Encapsulating unnatural sugars in chitosan nanoparticles, liposomes, and micelles, allows them to accumulate in tumor tissue via the EPR effect.^84,89–91,113^ Cheng et al. created an ultrasound-responsive liposome to improve the accumulation of Ac_4_ManNAz in tumors.^114^ They combined Ac_4_ManNAz-loaded liposomes with microbubbles (MBs), which stabilized the structure of liposomes in the bloodstream. MBs increased the size of liposomes, making them difficult to penetrate normal tissue blood vessels but easy to enter tumor tissues thus lowering Ac_4_ManNAz accumulation in normal organs. When tumor tissues were subjected to high amplitude ultrasound pressure, the MBs ruptured and released liposomes, allowing Ac_4_ManNAz to enter cells. However, due to the poor water solubility of Ac_4_ManNAz, these methods of encapsulating unnatural sugars may result in early release in the bloodstream. To address the issue, Lee et al. coupled Ac_3_ManNAz with poly(amidoamine) (PAMAM) dendrimer to create nano-sized metabolic precursors (Nano-MPs).^115^ Unnatural sugars attached stably to the nanoparticles due to covalent binding, decreasing their release into blood circulation. In the acidic TME, PAMAM can be digested, releasing Ac_3_ManNAz into tumor tissues. Because of their smaller size (10–20 nm), Nano-MPs are more easily endocytosed by tumor cells than Ac_4_ManNAz encapsulated nanoparticles (larger than 100 nm). Wang et al. also created azido-sugar conjugated glycopolyester NPs (GP-NPs) by initiating ring-opening polymerization (ROP) of 5-(4-(prop-2-en-1-yloxy) benzyl)-1,3-dioxolane-2,4-dione (allyl (all)-OCA).^73^ When compared to encapsulating unnatural sugars into nanocarriers, GP-NPs maintained the pharmacokinetic properties of metabolic precursors, delayed their release rates, and reduced their loss. Together, nanocarriers can deliver metabolic precursors to tumor tissues via EPR effects. With the advancement of nanotechnology, more efficient and tumor-selective nanocarriers will be developed to improve the tumor selectivity of metabolic precursors.

##### Chemical modification of metabolic precursors to target tumor

Bioorthogonal metabolic labeling of tumor cells can be increased by modifying unnatural sugars with chemical groups to target tumor cells or respond to TME. One strategy is to conjugate unnatural sugars with tumor-targeting ligands, such as folate.^116^ However, metabolic precursors are still endocytosed by normal cells. Therefore, researchers link tumor-specific response groups with metabolic precursors, which can only be activated in tumor tissues with specific agonists. For example, an Ac_3_ManNAz analog containing prostate-specific antigen (PSA) can only be absorbed by prostate cancer cells where PSA is presented.^117^ Kim et al. also created a cathepsin B-responsive Ac_3_ManNAz analog for bioorthogonal metabolic labeling of cathepsin B-overexpressing tumor cells.^87^ Cheng et al. developed a dual-enzyme-responsive metabolic precursor (DCL-AAM) that can only be used by tumor cells expressing both histone deacetylase (HDAC) and cathepsin L (CTSL).^98^ Cells lacking one or more of the enzymes could not be labeled, indicating increased tumor selectivity of metabolic precursor. While chemical-modified metabolic precursors are only activated and used by tumor cells, this technique has the potential to change the pharmacokinetic feature of metabolic precursors, hence affecting their clearance from circulation.^98^ As a result, their pharmacokinetics and stability should be considered after modification.

### Bioorthogonal metabolic labeling of pathogenic microorganisms

Bacteria and viruses pose a major threat to human health. Therefore, it is critical to developing DDSs that can accurately target pathogenic microorganisms and improve drug efficacy. Bioorthogonal metabolic labeling can be used to achieve in vivo tracing and targeted drug delivery of pathogenic microorganisms, which is of great significance for the development of efficient therapeutic regimens. Due to differences in architecture and components between pathogenic microorganisms and mammalian cells, there are certain disparities in the bioorthogonal metabolic labeling of bacteria and viruses. Bacterial polysaccharides are structures peculiar to bacteria that are engaged in pathogenesis. The imaging of bacterial invasion behavior is made possible by bioorthogonal metabolic labeling of bacterial polysaccharides.^118^ Swarts et al. produced a series of N_3_-modified trehalose analogs to deliver N_3_ onto the bacterial surface via the cell wall glycolipid synthesis pathway.^119^ The alkynyl-modified fluorescent probes were then coupled via a bioorthogonal reaction for subsequent glycolipid distribution and metabolite analysis. Additionally, researchers discovered that D-amino acids are essential components of bacterial cell walls but are lacking in eukaryotic cells.^120^ Therefore, N_3_-modified D-amino acid can be used to selectively label bacteria, allowing for the imaging of bacterially infected areas as well as targeted drug delivery.

Aside from bacteria, researchers also achieved bioorthogonal metabolic labeling of viruses. Viruses must be parasitized in living cells to survival. The genetic material of many viruses (for example, influenza virus and SARS-CoV-2) is amplified in living cells before being ejected by envelope action to generate progeny viruses.^121^ The viral envelope is derived from the host cell membrane, which allows bioorthogonal groups on the host cell membrane to be coated around the virus. Recently, bioorthogonal viral modification is accomplished via N_3_-labeled host cell membranes.^106,122,123^ This virus labeling approach is straightforward to implement and it can best preserve the structure and biological activity of the virus. It is hoped that this viral labeling technique may be used to administer antiviral drugs or to improve immune cells’ ability to recognize the virus, providing humans with an efficient weapon against virus infection.

## Application of bioorthogonal chemistry in targeted delivery

With the development of nanomedicines, biological ligands such as antibodies, polypeptides, and aptamers are typically bonded to the surface of nanoparticles to boost their binding to specific cells, resulting in targeted delivery.^124–126^ However, these targeting strategies complicate the preparation procedure and their targeting is largely dependent on differences in endogenous protein levels between target cells and normal cells.^127,128^ Recently, bioorthogonal chemistry has been widely exploited in the targeted delivery of therapeutic agents, providing a simple and effective technique for improving their targeting properties which increases their clinical application potential (Table 1).

**Table 1:** Summary of bioorthogonal chemistry in targeting delivery of nanomedicines

Targeting sites	Labeling agent	Labeled cell	Targeted agent	Targeted area	Therapeutic efficiency	Targeting efficiency	Ref
Tumor	Ac_4_ManNAz-loaded CNP	A549 tumor cell	BCN-Ce6-CNP	Subcutaneous tumor	Enhancing the effect of PTT	1.5-fold	^90^
	Ac_4_ManNAz-LP	A549 tumor cell	DBCO-ZnPc-LP	Subcutaneous tumor	Enhancing the effect of PTT and PAT	4.0-fold	^113^
	Ac_4_ManNAz	MCF-7 tumor cell	DLQ/DZ	Subcutaneous tumor	Enhancing the effect of Chemo-photothermal synergistic treatment	4.6-fold	^129^
	pHLIP-Tz (pTz)	HeLa tumor cell, VEC, TAF	TCO-HSA-ICG NP (THI)	Subcutaneous tumor	Improving tumor penetration of nanomedicine	2.6-fold	^131^
	Anti-CD11b-TCO	CD11b^+^ cell	MSNs-Tz	Subcutaneous tumor	Improving tumor penetration of nanomedicine	~2.2-fold	^133^
	Ac_4_ManNAz-loaded NP	4T1 tumor cell	DBCO-Ce6	Subcutaneous tumor	Improving tumor penetration of nanomedicine	*n.a*.	^136^
	iPDN_N3_	perivascular tumor	iPDN_DBCO_	Subcutaneous tumor	Improving tumor penetration of nanomedicine	~2.0-fold	^139^
	TK-PAMAM_PR104A_-N_3_	perivascular tumor	NP_Ce6_-DBCO	Subcutaneous tumor	Improving tumor penetration of nanomedicine	~1.5-fold	^140^
	Tumor acidity-activatable pre-targeted NPs	perivascular tumor	POLY-PROTAC	Subcutaneous tumor	Prolonging tumor retention of nanomedicine	~1.9-fold	^141^
	DA-Cys-PEG-*b*-PLA NPs	perivascular tumor	CBT-PEG-*b*-PLA NPs	Subcutaneous tumor	Prolonging tumor retention of nanomedicine	~2.5-fold	^142^
	Ac_4_ManNAz	B16F10 tumor cell	DBCO-aPDL1 aptamer	Subcutaneous tumor	Improving ICB efficacy	~3.0-fold	^144^
	Ac_4_ManNAz	CT26 tumor cell	PSQPNs-DBCO	Subcutaneous tumor	Enhancing the effect of NIR-II FI and PTT	~3.0-fold	^148^
	Ac_4_ManNAz	HEK293 cell	DBCO-UCNs	zebrafish	Modulating Ca^2+^ ion channel activity	~2.0-fold	^152^
	Ac_4_ManNAz	4T1 cell	Hf-AIE-PEG-DBCO	Subcutaneous tumor	Enhancing the effect of RDT	~3.0-fold	^158^
	CC49-TCO mAb	LS174T tumor cell	^212^Pb-DOTA-Tz	Subcutaneous tumor	PRIT	*n.a*.	^161^
Immune system	DSPE-PEG-N_3_	LEC	DL-O/IC	Lymph nodes	Improving LN targeting and retention time of vaccine	~3.0-fold	^109^
	Ac_4_ManNAz NP-loaded gel	DC	DBCO-IL-15/IL-15Rα, DBCO-OVA, DBCO-CpG	DC in vivo	Improving DC targeting of antigen, adjuvant, and cytokine	~3.0-fold	^111^
	Ac_4_GlcNAz	T cell	PEI-DBCO/Lentivirus Nanocomplex	T cell	Promoting gene transduction between lentivirus and T cells	~3.0-fold	^173^
Bacteria	D-AzAla@MIL-100 NP	Methicillin-resistant staphylococcus aureus (MRSA)	AIE NP-DBCO	MRSA	Specifically targeted elimination of bacteria	~2.5-fold	^176^
	d-Lys-PEG-TCO	Different kinds of bacteria	Tz-Au/MFO nanoparticle	Bacteria	Specifically targeted elimination of bacteria	*n.a*.	^177^
	D-Ala-N_3_	Different kinds of bacteria	Fluorescence turn-on probe	Bacteria	Discrimination and precise ablation of bacterial pathogens	*n.a*.	^179^
	D-Ala-N_3_	Gut bacteria	DBCO-probiotics	Gut bacteria	Modulating microbiome compositions	~6.0-fold	^182^

*Ac*_*4*_*ManNAz* N-azidoacetylmannosamine, *Ac*_*4*_*GlcNAz* N-azidoacetylglucosamine, *BCN* bicyclononyne, *DBCO* dibenzyl cyclooctyne, *Tz* tetrazine, *TCO* trans-cyclooctene, *CNP* chitosan nanoparticles, *LP* liposomes, *VEC* vascular endothelial cell, *TAF* tumor-associated fibroblast, *DC* dendritic cell, *PTT* photothermal therapy, *PAT* photoacoustic therapy, *ICB* immune checkpoint blockade, *RDT* radiodynamic therapy, *PRIT* pre-targeted radioimmunotherapy, *LN* lymph node, *n.a*. not applicable

### Targeted delivery of antitumor nanomedicine to tumor tissue

**Fig. 3:**
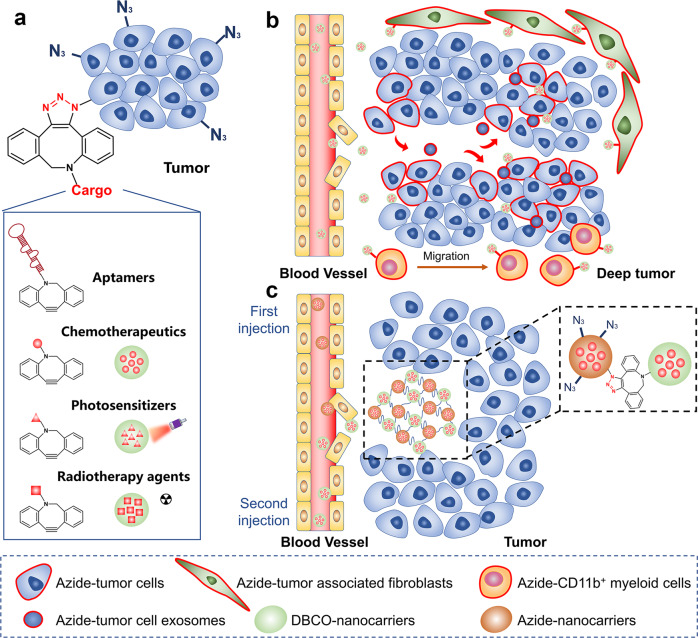
Targeted delivery of anticancer therapeutics to tumor tissues via bioorthogonal chemistry. **a** Cancer-targeted delivery of therapeutics. **b** Increased intratumorally penetration of nanoparticles in three ways: 1. Labeling tumor-associated fibroblasts; 2. Tumor cell exosomes; 3. Migration of immune cells. **c** Construction of drug reservoirs in tumor tissues via bioorthogonal chemistry

Tumor-targeting can be improved by modifying targeting ligands on the surface of nanoparticles. However, this approach is only effective for certain types of cancers, and the response rate varies between people. Bioorthogonal metabolic labeling introduces plenty of target sites on the surface of tumor cells that are not restricted by cell phenotype. Once tumor cells have been labeled with bioorthogonal groups (such as N_3_), drug-loaded nanoparticles with paired groups (such as DBCO) will target tumor cells selectively via bioorthogonal reaction. This tumor-targeting strategy is now being wildly studied for the targeted delivery of anticancer nanomedicines (Fig. 3a). For example, Kim et al. intravenously administered Ac_4_ManNAz-encapsulated chitosan nanoparticles (CNP) into A549 tumor-bearing mice to introduce N_3_ onto tumor cells.^90^ Following that, BCN-modified Ce6-loaded CNPs were injected and accumulated in the tumor via in vivo bioorthogonal reaction, which increases photothermal therapy (PTT) efficacy. After that, Xing et al. created tumor-targeting nanomicelles (DBCO-ZnPc-LPs) for the combination treatment of PTT and photoacoustic therapy (PAT).^113^ Ac_4_ManNAz-loaded nanomicelles (Ac_4_ManNAz-LPs) were delivered intravenously to cover tumor tissues with N_3_ groups. DBCO-ZnPc-LPs then accumulated in N_3_-labeled tumors due to a bioorthogonal reaction. Compared to control groups (tumor cells without N_3_ labeling), DBCO-ZnPc-LPs showed higher tumor selectivity and accumulation (~4.0-fold than the control group), inducing enhanced PTT and PAT under local laser excitation and ultrasound therapy, efficiently killing tumors and inhibiting tumor recurrence. Likewise, Qiao et al. developed a nanocomposite system (DLQ/DZ) based on DBCO-modified low-molecular-weight heparin-quercetin conjugate (DLQ) to deliver doxorubicin (DOX) and zinc phthalocyanine (ZnPc) for chemo-photothermal therapy.^129^

Although a great number of nanomedicines have been produced for the treatment of solid tumors, the tumor penetration depth of nanomedicines is limited due to the complex TME, resulting in poor therapeutic effects (Fig. 3b). Therefore, nanoplatforms capable of delivering nano drugs deep into tumors are required. Given that pH (low) insertion peptide (pHLIP) has a superior ability to enter membrane bilayers in acidic environments,^130^ Xie et al. developed a selective delivery strategy targeting acidic solid tumors using iEDDA reaction.^131^ Tz groups were first conjugated to pHLIP before being injected into mice. Tz-conjugated pHLIP (pTz) then entered the tumor, assisted by the acidic TME, and attached Tz onto the cell surface via membrane insertion. Because of the significant membrane-penetrating action of pHLIP,^132^ 44.0% of vascular endothelial cells (VECs) and 50.5% of tumor-associated fibroblasts (TAFs) were labeled with Tz. This resulted in more targeting sites for nanomedicines. Following that, they constructed indocyanine green (ICG)-encapsulated TCO-modified albumin nanoparticles (THI) and folic acid-modified nanoparticles (FHI) as a control. The results revealed that pTz/THI group exhibited more aggregation in tumor tissue than the FHI group (~ 2.6-fold). As a result of labeling three types of cells in tumor tissues, this DDS improves drug penetration depth and accumulation in tumors, providing new strategies for cancer therapy.

Being inspired by the idea that inflammatory immune cells could be recruited to promote tumor growth, Hyeon et al. developed a click reaction-assisted immune cell targeting (CRAIT) strategy to deliver nanomedicines to the inside of tumors.^133^ TCO was linked to CD11b antibody (anti-CD11b-TCO) and Tz was conjugated to DOX-loaded mesoporous silica nanoparticles (MSNs-Tz). Anti-CD11b-TCO bound to CD11b^+^ myeloid cells in tumor tissue and improved the coupling of MSNs-Tz to CD11b^+^ myeloid cells via bioorthogonal reaction. As an active transporter, CD11b^+^ cells demonstrated significant motility in tumor tissue and were capable of transporting DOX-loaded nanoparticles deep into the tumor. The findings showed that CD11b^+^ cell-mediated delivery had a more uniform distribution and deeper tumor penetration than passive targeting approaches, with a 2-fold increase of nanoparticles in avascular tumor regions.

Except for the employment of inflammatory cells and extracellular matrix cells in tumor tissues, the direct use of tumor cells’ extracellular vesicles (EVs) can also improve tumor penetration of nanomedicines.^134,135^ Wang et al. created a tumor EV-mediated bioorthogonal labeling approach to deliver drug delivery platforms into the deeper tumor.^136^ Azide sugar nanoparticles (Az-NPs) labeled perivascular tumor cells with a high number of N_3_ groups. N_3_ groups were then distributed throughout the tumor tissue via N_3_-labeled EVs, leaving the entire tumor area well-labeled. The results revealed that DBCO-Ce6 selectively penetrated tumor tissue by bioorthogonal reaction and induced photodynamic therapy (PDT). This tumor EV-based bioorthogonal drug targeting technology provides a novel alternative strategy for improving the tumor selectivity and permeability of DDSs.

Studies have shown that even though NPs allow more drug delivery to the tumor, only a small fraction of the drug can ultimately be retained at the tumor site. Therefore, developing methods to enhance the retention of nanomedicines in the tumor region would enhance the antitumor potential of nanomedicines.^137,138^ Bioorthogonal chemistry has been explored to couple nanoparticles to form a micron-level drug reservoir in tumor tissues, thereby improving tumor targeting and intratumor retention of antitumor drugs (Fig. 3c). In general, drug-free nanoparticles enter the tumor tissue through the EPR effect and expose pre-shielded bioorthogonal groups in TME. Then drug-loaded nanoparticles are injected after a while to bind to drug-free nanoparticles in the tumor via bioorthogonal reactions to form a large-size drug retention system. For example, Yuan and co-workers created a DDS based on the tumor acidic environment and bioorthogonal chemistry to improve DOX and NO donor accumulation and penetration in tumor tissues.^139^ PAMAM was used to link DOX and NO donors to produce polymeric nanoparticles (PDNs). iCPDNs were produced by combining tumor acid-responsive cross-linker maleic acid amide with PDNs. iCPDNs were then modified with N_3_ and DBCO to generate iCPDN_DBCO_ and iCPDN_N3_, with DBCO active site masked by poly (2-azetidinyl methacrylate) (PAEMA). Because of PAEMA’s fast acidic response rate in tumors, DBCO was immediately exposed, and the accumulation of iCPDN_N3_ in tumor tissue was attracted by an effective bioorthogonal reaction, generating a large-sized drug reservoir. In an acidic environment, maleic acid amide slowly dissociated, releasing DOX and NO donors that allowed them to penetrate deep into the tumor. NO reduced HIF-1α levels, reversed tumor hypoxia tolerance, improved the chemotherapeutic efficacy of DOX, and boosted the antitumor immune response. Meanwhile, they created a size-variable nano-drug based on bioorthogonal reaction for the combination of PDT and hypoxia-activated prodrugs (HAP).^140^ DBCO was modified with poly(2-(hexamethylene) ethyl methacrylate)-b-poly(ε-caprolactone) (DBCO-PC7A-PCL) before being loaded with Ce6 to create NP Ce6-DBCO with ultrafast pH-responsive reactivity via self-assembling. They also constructed ROS-cleavable HAP nanoparticles (TK-PAMAMPR104A-N_3_) that were N_3_-modified. NP_Ce6_-DBCO can immediately respond to the acidic environment and expose DBCO while slowly releasing Ce6, thereby attracting TK-PAMAM_PR104A_-N_3_ to the tumor tissue via bioorthogonal chemistry. Ce6 promoted the generation of high-level ROS which aggravated tumor hypoxia, prompted the cleavage of TK-PAMAM_PR104A_-N_3_, activated HAP, and destroyed the tumor when exposed to 660 nm laser irradiation. These nanoparticles based on tumor acid environment response and bioorthogonal reaction can not only improve the targeted accumulation of drugs in tumor tissues but also build drug reservoirs to realize the continuous release of therapeutics.

In addition, Yu et al. used this in situ bioorthogonal tumor targeting strategy to design a poly-PROTAC (POLY-PROTAC) nanoplatform for targeted degradation of BRD4, which improved tumor selectivity and precise delivery of proteolysis targeting chimeras (PROTACs).^141^ DBCO-modified POLY-PROTAC achieved selective tumor targeting and retention by bioorthogonal reaction with N_3_-modified self-assembled micelles that enter the tumor beforehand, resulting in an approximately 1.9-fold increase in nanoparticle accumulation in the tumor compared to the group without bioorthogonal reaction. Yang et al. also constructed an in situ self-assembled drug reservoir using bioorthogonal reactions between cyanide of cysteine (Cys) and 2-cyanobenzothiazole (CBT) for the resident and sustained release of multiple drugs, improving the performance of a cocktail of chemoimmunotherapies.^142^ These studies illustrate that bioorthogonal chemistry can not only improve the tumor selectivity of drugs but also prolong the residence time of drugs in the tumor and transport them to the precise site of action. While combined with external stimuli such as light, heat, and magnetic fields, controlled drug release and deep tumor penetration are achieved, providing a solution to overcome drug resistance and enhance anti-tumor immune response.

In addition to demonstrating strong efficacy in chemotherapy, PDT, and PTT, bioorthogonal chemistry has also been used in a variety of novel oncology treatment strategies to improve tumor selectivity and specific targeting.

Aptamer, a single-stranded DNA or RNA oligonucleotide, has strict recognition and high affinity for the target molecule.^143^ Compared with antibodies, it has better specificity and lower immunogenicity. However, the affinity of current aptamers is not as good as that of antibodies due to poor preparation conditions. Given this, Tan et al. combined the aptamer recognition ability with N_3_/DBCO bioorthogonal chemistry to achieve logic-driven immune checkpoint blockade (ICB) therapy.^144^ They chose the anti-PDL1 (aPDL1) aptamer as a model and attached DBCO to the 5’ end of the aptamer. N_3_ acted as a “chemo-receptor” binding to cancer cell surface glycoprotein, allowing the ICB aptamer to covalently bind on the cancer cell surface for a long time without detachment and internalization, improving the efficiency and accuracy of cancer therapy by blocking the PD1-PDL1 signaling pathway. By manipulating the aptamer-based logical response to achieve selective recognition and coupling of aPDL1 agents on target cells, this strategy ameliorates the shortcoming of aPDL1 antibodies that do not work long-term and improves the efficiency of ICB at the molecular level.

The 1064 nm activatable NIR-II FI/PTT nanoplatform has the potential for accurate cancer diagnosis and effective PTT therapy.^145,146^ However, fluorescence emission competes with photothermal inactivation.^147^ Therefore, developing a perfect 1064 nm activatable nanoplatform with both a strong NIR-II FI signal and high PTT efficiency is expected. Fan et al. created a square amine-based semiconducting polymer dye (PSQPNs-DBCO) for accurate NIR-IIa fluorescence imaging and enhanced PTT treatment.^148^ Tumor cells were pretreated with Ac_4_ManNAz to label them with N_3_ groups, which led to the selective accumulation of PSQPNs-DBCO in tumor tissues. Under 1064 nm excitation light, PSQPNs-DBCO induced strong PTT to kill tumor cells. As a result, PSQPNs-DBCO provides a light excitable therapeutic nanoplatform indicating substantial clinical translation potential.

Light-gated ion channels, as opposed to photosensitizers that generate PDT and PTT, can alter cellular activity both spatially and temporally by guiding ion transport across the cell membrane.^149^ They are regarded as a tool for enhancing therapeutic efficacy and personalizing treatment. However, most light-gated channels are activated by ultraviolet (UV) or visible light, which can cause cell damage and limited light penetration.^150,151^ To solve the problem, Xing et al. constructed a bioorthogonal-based NIF-triggered approach for modulating Ca^2+^ ion channel activity.^152^ First, the cell membrane was labeled with N_3_ groups. Then, DBCO-modified neodymium (Nd^3+^)-doped upconversion nanocrystals (DBCO-UCNs) were selectively linked to cells via a bioorthogonal reaction, which could convert 808 nm NIR light to 480 nm light. The protein channelrhodopsins-2 (ChR2) was triggered by 480 nm light, allowing for control of Ca^2+^ inflow. DBCO-UCNs controlled Ca^2+^ ion channel activity and enhanced caspase-3 expression in zebrafish, showing that this technique was likely to trigger apoptosis. If this bioorthogonal-based light-controlled ion channel regulation mechanism can be applied to tumors, it is predicted to yield a new method for cancer treatment.

Radiodynamic therapy (RDT) is a novel cancer treatment approach that uses ionizing radiation to generate local PDT, overcoming the constraints of PDT penetration depth and traditional radiotherapy (RT) energy concentration.^153^ Nanoscale metal-organic frameworks (NMOFs) consisting of photosensitizers may directly transport X-rays to photosensitizers and activate them to realize RDT via inelastic scattering of photoelectrons.^154,155^ However, due to flaws such as quenching the ACQ effect, NMOFs have not attained the anticipated therapeutic impact.^156,157^ Liu et al. used AIE PS and hafnium ions (Hf^4+^) to construct an NMOF (Hf-AIE) for synergistic RDT and RT.^158^ The DBCO/N_3_ bioorthogonal reaction was exploited to increase NMOF tumor selectivity. Hf-AIE-PEG-DBCO nanoparticles were anchored to N_3_-labeled tumor cells via bioorthogonal reactions. The anticancer effect was satisfactory due to the profound tissue penetration of X-rays, even with 3 mm of lean pork covering the tumor. As a result, this bioorthogonal NMOF offers considerable potential for cancer therapy.

Unlike RDT, radioimmunotherapy (RIT) is a one-step procedure in which tumor-targeting monoclonal antibodies (mAbs) are radiolabeled.^159^ However, the poor pharmacokinetics of mAbs have hampered RIT. Pre-targeted radioimmunotherapy (PRIT) is a multi-step method that separates the administration of tumor-targeting molecules from the delivery of radionuclides, allowing for high doses of radiation with minimum exposure to normal tissue.^160^ Quinn et al. tested the radiolabeling efficiency and pharmacokinetic features of two Tz-PEG chelators based on ^212^Pb (DOTA-PEG_10_-Tz and TCMC-Bn-SCN-PEG_10_-Tz).^161^ They created CC49-TCO and used the following three-step pre-targeting strategy in the subsequent RIT: 1. Pre-targeting CC49-TCO; 2. Scavenger injection; 3. ^212^Pb-DOTA-Tz injection. PRIT inhibited tumor development and enhanced survival in the LS174T model, demonstrating good therapeutic benefits and intriguing clinical prospects.

### Targeted delivery of immunomodulator to immune cells or immune organs

Immunotherapy is one of the ways to treat cancer, by activating or strengthening the anti-tumor immune response. Currently, bioorthogonal chemistry is used for lymph node (LN) targeting of cancer nanovaccine, targeted delivery of immunotherapeutics to dendritic cells, and targeted delivery of lentivirus to T cells to enhance the anticancer effect.

**Fig. 4:**
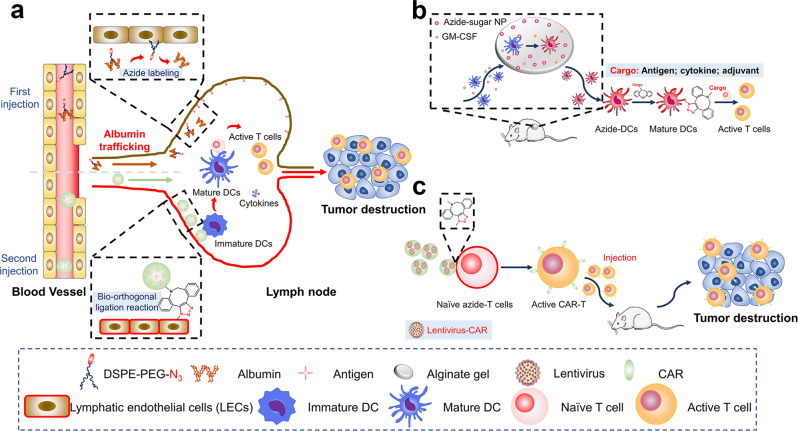
Targeted delivery of immunomodulators to immune cells or immune organs. **a** Lymph node targeting of nanovaccine. **b** In situ DC targeting of antigen, cytokine, and adjuvant. **c** T cell targeting of lentiviral transfection

Nanovaccine employs nanoparticles as carriers to deliver specific antigens and adjuvants.^162–164^ Because of the size advantage (10–100 nm), nanovaccine is simpler to accumulate in LNs.^165–168^ Furthermore, because nanovaccine is comparable in size to pathogens, it is easily taken up by antigen-presenting cells (APCs), delivering antigens and immune stimulators to targeted immune cells. Tumor-associated antigen (TAA)-specific T cells can be triggered directly in this process to drive adaptive immune response and destroy tumor cells. However, the lymphatic system is a unidirectional circulatory network that finally drains into the bloodstream.^169,170^ If nanoparticles are not taken up by APCs in time, they will be expelled from LNs by lymph fluid and enter the blood circulation for clearance or uptake by macrophages. Therefore, Nie et al. created a nanovaccine that selectively targeted LNs utilizing bioorthogonal chemistry to extend its residence time in LNs (Fig. 4a).^109^ DSPE-PEG-N_3_ was injected subcutaneously and entered LNs via the intrinsic transport mechanism of albumin and then embedded in the cell membrane via lipid insertion. The findings revealed that little N_3_ was displayed on the surface of APCs, whereas around 90% of lymphatic endothelial cells (LECs) were labeled with N_3_ groups. LECs are found in lymph node sinus, subcapsular sinus (SCS), and medullary sinus (MS), which form the channel for nanovaccine to enter and exit LNs. These N_3_-labeled LECs provided plenty of targets for LN targeting. DBCO-modified nanovaccine (DL-O/IC) loaded with OVA257-264 peptide and TLR agonist poly(I:C) targeted to N_3_-labeled LECs and absorbed by them. This LN targeting method based on bioorthogonal chemistry is classified as an active lymph node accumulation system (ALAS). ALAS increased the accumulation and residence time of nanovaccine in LNs compared to the control group without DSPE-PEG-N_3_ injection (NLAS). Around 85% of APCs took up DL-O/IC (~50% of APCs in NLAS). In vivo studies revealed that ALAS stimulated the antitumor immune response and prevented lung metastases of mice melanoma. This bioorthogonal chemistry-based LN targeting strategy boosts selectivity and accumulation of nanovaccine in LNs while increasing antigen and adjuvant uptake by APCs, resulting in great anticancer effects. ALAS will provide a potent platform for individualized immunotherapy by combining the delivery of tumor antigens and immune adjuvants. Meanwhile, the combination of immune checkpoint inhibiting antibodies is predicted to boost the therapeutic effect of ALAS against cancer.

In addition to improving LN targeting, bioorthogonal chemistry can be employed for dendritic cell (DC) targeting of immunological stimuli (such as antigens and immune agonists) (Fig. 4b). Mooney et al. used bioorthogonal chemistry to construct an immunotherapeutic strategy by directly altering DC activity in vivo.^111^ They stored Ac_4_ManNAz-loaded NPs in porous alginate gels and injected them subcutaneously into mice. At the injection site, granulocyte-macrophage colony-stimulating factor (GM-CSF) was released to recruit DCs in vivo. The Ac_4_ManNAz-loaded nanoparticles were then taken up by DCs and N_3_ groups were introduced onto the DC membrane. About 22% of DCs within the scaffolds were N_3_-labeled, which was much greater than other controls. DBCO-IL-15/IL-15R was then injected into mice to activate DCs via bioorthogonal reaction. The results revealed that DBCO-IL-15/IL-15R infusion increased the proliferative activity of CD8^+^ T cells. When paired with a vaccine containing neoantigens M27 and M30, 25% of the mice showed total tumor regression and a 57% improvement in median survival. The ability of N_3_-modified DCs to uptake DBCO-OVA and DBCO-CpG was also tested. The proportion of DBCO-OVA and DBCO-CpG-targeted DCs, as well as the number of OVA-specific CD8^+^ T cells, were higher in N_3_-labeled DCs compared to unlabeled DCs. This straightforward in vivo DC targeting method increases antigen, adjuvant, and cytokine delivery to DCs, which provides a reference for bioorthogonal metabolic labeling of macrophages and other types of immune cells in vivo.

In addition, bioorthogonal chemistry can improve lentivirus targeting to T cells. Adoptive T cell immunotherapy, such as CAR-T and T cell receptor T cell (TCR-T) therapy, has emerged as an effective therapeutic method.^171^ However, genetic engineering of naive T cells during production is still ineffective. Although viral transduction is the most used route of gene delivery, it only generates 4–30% positive T cells, thus reducing the efficacy of adoptive T cell treatment.^172^ So far, improving viral transduction efficiency to T cells while minimizing adverse effects is a major problem. In light of this, Cai et al. developed a method to improve the selectivity of lentiviral transfection via bioorthogonal chemistry (Fig. 4c).^173^ T cells were first modified with N_3_. Lentiviruses were then encased by PEI-DBCO which induced an efficient and selective bioorthogonal interaction with N_3_ on T cells, promoting gene transduction. The results demonstrated that the transduction efficiency of lentivirus was boosted by 20–80%, resulting in increased production of anti-CD19 CAR-T cells. This bioorthogonal chemistry-based targeting technique is simple, effective, and can be widely used in CAR-T generation.

### Targeted delivery of antibiotics to bacteria

**Fig. 5:**
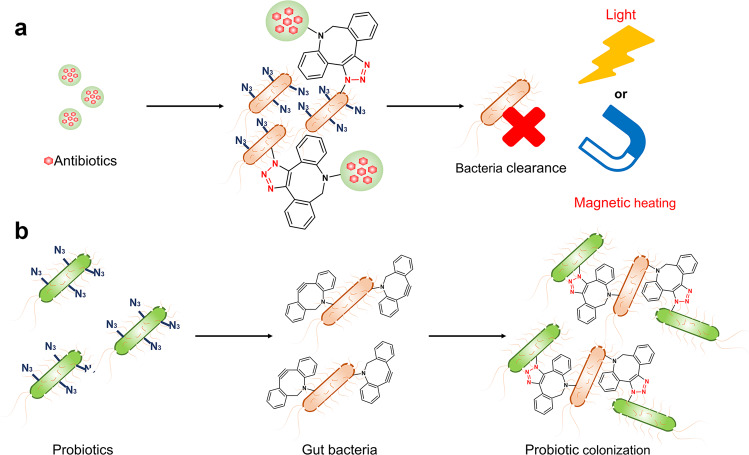
Targeted delivery of antibiotics to bacteria. **a** Antibiotic nanoparticles bind to the N_3_-labeled bacteria by bioorthogonal chemistry and exert bacterial killing under the action of light or magnetic field. **b** Improving probiotic colonization via bioorthogonal chemistry

Different antibiotics can selectively kill different types of bacteria. However, antibiotic overuse can result in bacterial resistance, making treatment of drug-resistant bacterial illnesses challenging.^174^ Although nanoparticles have been applied to detect and kill bacteria, identifying and targeting specific bacteria remains a difficult task.^175^ Therefore, alternative ways for detecting, targeting, and killing bacteria in a non-invasive manner are needed to avoid antibiotic resistance. Metabolic labeling technology can display bioorthogonal groups on the bacterial surface, which can be an effective way to boost the antibacterial effect of nanomedicines (Fig. 5a). Liu et al. devised an in vivo detection and therapy technique for bacteria.^176^ They created d-AzAla-loaded organic framework nanoparticles (d-AzAla@MIL-100(Fe)NPs) that could selectively accumulate in the infection site and release d-AzAla in an H_2_O_2_ inflammatory environment. Bacteria utilized d-AzAla to introduce N_3_ onto bacterial cell walls. Subsequent intravenous injections of DBCO-modified AIE nanoparticles resulted in specific tracking and efficient clearance of bacteria in infected tissues.

Meanwhile, Duan et al. applied bioorthogonal chemistry to label MnFe_2_O_4_/Au (MFO/Au) Janus magnetic nanoparticles on the surface of G^+^ bacteria, allowing them to be detected and killed.^177^ D-Lys-PEG-TCO was incorporated into peptidoglycan of G^+^ bacteria via the peptidoglycan production pathway by co-cultivation with bacteria. A bioorthogonal reaction was used to selectively bind the Tz-functionalized MFO/Au Janus magnetic nanoparticles to the cell wall. Bacteria linked to nanoparticles can be enriched by magnetic separation and killed in a high-frequency alternating magnetic field.

In addition to the above strategies, probes based on fluorophores with aggregation-induced emission (AIEgens) with photosensitizer properties can not only identify and label bacteria, but also irreversibly damage bacterial membranes by generating ROS.^178^ Liu et al. designed a bioorthogonal fluorescence turn-on probe TPEPA for identification and precise ablation of bacterial pathogens.^179^ TPEPA was expected to show weak fluorescence upon solubilization but turned-on fluorescence upon bioorthogonal click reaction between the alkyne fraction of TPEPA and azide groups on the surface of bacteria pretreated with N_3_-modified amino acid. Under fluorescence microscopy, TPEPA could clearly distinguish between G^+^ and G^−^ bacteria. In addition, TPEPA could produce ROS under white light irradiation, which implied its dual role as a selective imaging agent and phototherapeutic agent for light-guided ablation of bacteria with high selectivity and specificity.

There is growing evidence that probiotics can influence and modulate microbial fractions through oral delivery, which is considered a promising approach for the prevention and treatment of bacterial infections and intestinal diseases.^180^ Unfortunately, biological challenges such as low availability and inadequate retention in the gastrointestinal tract during oral delivery have largely limited the clinical translation of probiotics.^181^ In light of this, Zhang et al. reported a bioorthogonal-mediated probiotic delivery strategy for enhanced probiotic colonization in the intestinal tract (Fig. 5b).^182^ First, azide-modified D-alanine (N_3_-DAA) was metabolically bound to the peptidoglycan of the intestinal bacteria for azide decoration. Subsequently, DBCO-modified probiotics were orally administered to mice, and bioorthogonal reactions between probiotics and intestinal residents were performed to improve the adhesion and the colonization efficiency of probiotics. In a mouse model of dextran sodium sulfate (DSS)-induced colitis, bioorthogonal-mediated *Clostridium typhimurium* (*C. butyricum*) showed efficient colonization and effective therapeutic effects, which may be achieved by modulating the intestinal flora (reducing the abundance of harmful bacteria and increasing the abundance of beneficial bacteria). This bioorthogonal-mediated bacterial delivery strategy for intestinal flora regulation offers a promising strategy for the treatment of gastrointestinal diseases.

## Application of bioorthogonal chemistry in developing bio-inspired DDSs

Cell surface modification means modifying cell membranes through physical, chemical, or biological methods to get cells with stronger or new functionalities.^183–185^ Currently, cell surface modification consists of the following strategies: 1. Physical adsorption by electrostatic or hydrophobic interaction; 2. Chemical coupling via covalent bonding; 3. Modification of cell membrane via genetic or metabolic engineering; and 4. Deformation of cell membranes via electric fields. However, the above techniques have shortcomings in terms of cellular compatibility, target applicability, uniformity of distribution of modified molecules on the membrane surface, and residence time of modified molecules on the membrane surface.^186,187^ Recently, the application of bioorthogonal chemistry has resulted in safer and more precise cell surface modification. On the one hand, bioorthogonal chemistry is more stable, efficient, and non-toxic.^75^ On the other hand, this technique accurately regulates the functionalization of cell membranes thus creating opportunities to develop bio-inspired DDSs. The following section discusses the use of bioorthogonal chemistry in developing DDSs (Table 2).

**Table 2:** Summary of bioorthogonal chemistry in developing bio-inspired DDSs

Type of DDSs	Labeling agent	Labeled cell	Engineering modification	Purpose	Ref
Biomimetic DDSs	Ac_4_GalNAz	T cell	N_3_	Enhancing T cell recognition and cytotoxicity to tumor cell	^194^
	Ac_4_GalNAz	T cell	N_3_	Dual tumor targeting of T cell membrane tumor immune recognition and bioorthogonal reaction	^82^
	Ac_4_GalNAz	CAR-T cell	N_3_	Improving tumor immunotherapy of CAR-T cells	^195^
	Azide-Cho	leukocyte	DBCO-EpCAM	Detection and accumulation of CTCs	^196^
	Azide-Cho	macrophage	DBCO-GRD	Delivering siRNA to tumor tissues	^83^
	Ac_4_ManNAz	host cell	DBCO-heparin	Enhancing SARS-CoV-2 virus binding and neutralization	^201^
	Azide-Cho	T cell	DBCO-aCD28 and DBCO-pMHC-1	CTL activation and amplification	^274^
	Ac_4_ManNAz	DC2.4 cell	DBCO-aCD3ε	CTL activation and amplification	^110^
	Azide-Cho	leukocyte	DBCO-PD-1	Targeting tumor cells and regulating the tumor microenvironment	^208^
	Azide-Cho	cancer cell	DBCO-aCD205	Targeting DC cells in LNs	^205^
	Azide-Cho	macrophage	DBCO-aCD47 and DBCO-aSIRPα	Targeting tumor cells and regulating the tumor microenvironment	^211^
	DBCO-sulfo-NHS	neutrophil	N3@uPB	RA treatment	^212^
Cell-based DDSs	DSPE-PEG_5k_-Tre	T cell	BCN-Lipo-Ava	Enhancing T cell tumor killing ability	^108^
	Ac_4_GalNAz	CAR-T cell	indocyanine green nanoparticles (INP)	Enhancing CAR-T cell tumor killing ability	^225^
	Ac_4_GalNAz	CAR-T cell	IL-12 nanostimulant (INS)	Enhancing CAR-T cell tumor killing ability	^226^
	Ac_4_ManNAz	hMSC	BCN-CNP-Cy5.5	MSC tracking	^228^
	Ac_4_ManNAz	MSC	Paclitaxel-loaded DBCO-NPs	Paclitaxel tumor targeting	^229^
	Ac_4_ManNAz	NK	N_3_, IL-21 nanoparticles	Enhancing NK cells function and tumor targeting	^232^
	MPB-sia-α2-6-Lac-N_3_	NK9.2 cell	MPB-sia	Endowing NK cells with the ability to target CD22^+^ tumor	^234^
	Ac_4_ManNAz	CAR-T cell	Hyaluronidase and aPDL1	Enhancing CAR-T tumor penetration ability and therapeutic efficacy	^235^
	Ac_4_ManNAz	NIT-1 cell	PD-L1/CD86/Gal-9 DBCO-functionalized dendrimer	Stimulating the consumption of islet antigen-specific T cells and reversing T1DM	^236^
	Antibody clickers (ABCs)	HER2-overexpressing NIH3T3 cells	ABC-labeled trastuzumab	Regulating the physical properties of the antibody	^241^
	Ac_4_GalNAz	HSC	platelet–aPD-1 (P-aPD-1)	Harnessing the bone marrow-targeting ability of HSCs and the inflammation-specific activation of platelets to enhance aPD-1 treatment of AML	^242^
Bacteria-based DDSs	D-Ala-N_3_	*Escherichia coli* MG1655	cerium oxide nanoparticles	Integrating lead detoxification and ROS elimination	^244^
	TCO-vancomycin	G^+^ bacteria	GNP-Tz	Bacterial SERS imaging and antimicrobial PTT	^245^
Phage-based DDSs	l-azidohomoalanine	*F. nucleatum*	D-IDNP	Harnessing the bacterial targeting ability of phages to modulate gut microbiota for CRC	^249^

*Ac*_*4*_*ManNAz* N-azidoacetylmannosamine, *Ac*_*4*_*GalNAz* N-azidoacetylgalactosamine, *Azide-Cho* azide-modified choline, *MSC* mesenchymal stem cell, *hMSC* human mesenchymal stem cell, *HSC* hematopoietic stem cells, *CTC* circulating tumor cell, *CTL* cytotoxic T lymphocyte, *DC* dendritic cell, *LN* lymph node, *RA* rheumatoid arthritis, *T1DM* type 1 diabetes mellitus, *AML* acute myeloid leukemia, *CRC* chronic colon cancer

### Constructing biomimetic DDSs by bioorthogonal chemistry

**Fig. 6:**
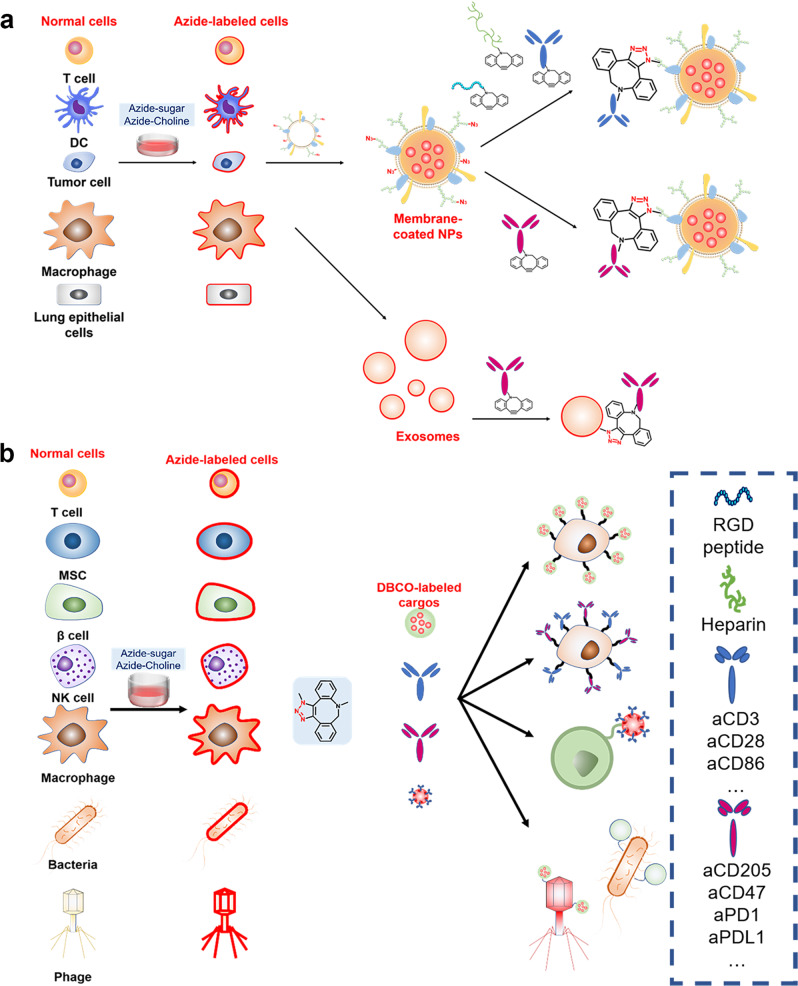
Application of bioorthogonal chemistry in developing bio-inspired DDSs. **a** Constructing biomimetic DDSs by bioorthogonal chemistry. Targeting and immunoregulating molecules can be modified onto cell membranes to construct multi-functional biomimetic DDSs. **b** DBCO-modified nanoparticles, antibodies, and platelets can be conjugated onto cell membranes or the surface of bacteria and phages to construct functionalized cell-based, bacteria-based and phage-based DDSs

Biomimetic techniques to enable precise drug delivery has become a research focus.^188–191^ The use of natural cell membranes to cover nanoparticles avoids immune system clearance while keeping innate targeting capabilities. The majority of the targeting effects of cell membrane-based biomimetic DDSs are mediated by cytokines or chemokines. These inducers bind to receptors on cell membrane surfaces, allowing biomimetic systems to target injured tissues. However, the cell membrane has limited functionalities, and the full surface coating of biomimetic nanoparticles is rare, which could reduce the delivery effect.^192^ Also, it is hard to precisely functionalize cell membranes without interfering with fundamental functions. Bioorthogonal chemistry provides an effective tool for arming biomimetic systems with targeted molecules and immune functional groups, providing biomimetic DDSs with increased targeting or immunoregulating capabilities (Fig. 6a).

#### Modification of targeting molecules

It is known that activated T lymphocytes can detect and kill tumor cells.^193^ However, tumor receptor heterogeneity always leads to poor effectiveness. Cai et al. created novel modified T cells (N_3_-T cells) to improve T cell recognition of tumor cells.^194^ BCN-tumor cells were coupled to N_3_-T cells in this targeting technique because of bioorthogonal reaction, which increased activate T cells to kill tumors. The findings showed that a rapid and effective bioorthogonal reaction on the cell surface boosted tumor targeting and enhanced T cell tumor-killing ability. On this basis, his team created ICG nanoparticles with N_3_-labeled T cell membranes (N_3_-TINPs) for PTT.^82^ Tumor cells were pre-labeled with BCN via intratumoral injection of Ac_4_ManN-BCN. Following that, N_3_-TINPs identified the natural antigens and BCN groups on tumors via immunological identification and bioorthogonal reactions, delivering ICG to tumor cells. The accumulation of N_3_-TINPs in tumors improved the efficacy of PTT. This dual tumor-targeting strategy based on tumor immune recognition and bioorthogonal reaction combine the strength of both single targeting strategies while overcoming tumor heterogeneity for biomimetic systems. Recently, they have applied N_3_/BCN bioorthogonal chemistry for CAR-T cell targeting, which improved CAR-T selective recognition and anticancer efficacy.^195^

Bioorthogonal reactions can also improve the targeting capacity of biomimetic systems by modifying specific ligands on the cell membrane. Xie et al., for example, developed a new biomimetic immunomagnet (IMS) to enrich circulating tumor cells (CTCs) using N_3_-modified leukocyte membranes.^196^ According to research, the lack of CTCs in blood makes it hard for efficient CTC enrichment or targeting.^197^ They used bioorthogonal reaction to attach epithelial cell adhesion molecule (EpCAM) to the surface of IMSs. EpCAM is a common cancer marker that is thought to be an excellent antigen for detecting CTCs. IMSs targeted EpCAM-positive CTCs, resulting in a huge number of IMSs covering the surfaces of CTCs. Using a magnetic field and microfluidic technology, almost 90% of CTCs could be extracted from whole blood in 15 min. This biomimetic system handles the problem of CTC enrichment, which is critical for cancer diagnosis and treatment. In addition, they developed a siRNA DDS based on N_3_-labeled macrophage membranes.^83^ By linking DBCO-GRD on macrophage membrane via bioorthogonal reaction, magnetic nanoparticles loaded with siRNA could be transported to tumor cells overexpressing RGD receptors under the action of a magnetic field.

In addition to tumor targeting, bioorthogonal functionalized biomimetic DDSs have been investigated for viral targeting and prevention. In 2020, a “cellular nanosponge (NS)” was created using cell membranes obtained from human lung epithelial cells or macrophages to prevent SARS-CoV-2 infection.^198^ Notably, these NSs are home to a variety of protein receptors (including the cellular angiotensin-converting enzyme 2 receptor, which binds to the SARS-CoV-2 S protein). In terms of SARS-CoV-2, these biomimetic nanoparticles are identical to normal cells, making it difficult for the virus to distinguish them from common cells. As a result, if a substantial dose of NSs is administered, the virus can be “sucked away” by NSs, lowering the proportion of normal cells infected by the virus. Further research revealed that S protein interacts with cell surface glycosaminoglycans like heparin preferentially.^199^ This contact transformed S protein to an open shape, which improves binding to angiotensin converting enzyme 2 (ACE2).^200^ In light of this, Zhang et al. utilized bioorthogonal reaction to link DBCO-modified heparin on N_3_-modified NS to produce heparin-modified cellular nanosponges (HP-NS).^201^ Compared to NS, the binding capacity of HP-NS to S protein was raised by around 5.4 times. In the virus neutralization experiment, HP-NS demonstrated a higher inhibitory effect on virus infection, with an IC50 value of 0.0254 g/ml, compared to 170 g/ml for NS. The results indicated that heparin modification increased the inhibitory effect on SARS-CoV-2, highlighting the broad application potential of bioorthogonal functionalized biomimetic systems in viral prevention.

#### Modification of immunoregulating molecules

Immune cells have a crucial role in antitumor immunotherapy. Immune cells communicate with one another via ligands and receptors on the cell membrane. T cells, for example, can only activate, proliferate, differentiate, and kill tumor cells when stimulated by specific ligands on DCs.^202^ However, tumor cells have evolved numerous defense mechanisms to avoid T cell recognition. For example, highly expressed PD-1 on tumor cells binds to PD-L1 on T cells, limiting T cell cytotoxicity. Antibodies that boost immune cell activity or decrease immune evasion can be modified on biomimetic DDSs through bioorthogonal reactions, which co-regulate antitumor immune response with drugs or immune adjuvants in nanoparticles, resulting in improved antitumor effects.

For example, Xie et al. created a magnetic resonance-guided artificial antigen-presenting cell (aAPC) for T cell activation.^201^ These aAPCs are based on SIINFEKL peptide-loaded magnetic nanoparticles that major histocompatibility complex (pMHC-I) and anti-CD28 were decorated to the N_3_-engineered leucocyte membrane through the bioorthogonal reaction between N_3_ and DBCO. In vitro experiments revealed that aAPCs could bind to the surface of T cells in the presence of pMHC-I and anti-CD28, thereby activating CD8^+^ T cells. After intravenous injection, many aAPCs retained their significant T-cell binding capabilities. This highly effective cell-cell interaction created the necessary conditions for Cytotoxic T lymphocytes (CTL) infiltration into tumor tissue via magnetic resonance imaging (MRI). However, such aAPCs only activate single antigen-specific T cells, not T cells targeting numerous tumor antigens. At the same time, the aAPCs ignore phagocyte phagocytosis. If macrophage phagocytosis can be exploited, it is expected that aAPCs will have increased immune activation capacity. In light of this, Li et al. presented better aAPCs for linking DBCO-modified CD3 antibody to N_3_-labeled DC membranes and coating PLGA nanoparticles with imiquimod.^110^ These aAPCs not only retained almost all of the CD28 and MHC I-Ag on the cell membrane but also improved the interaction with T-cell CD3 receptors via CD3 antibody, facilitating the accumulation and retention of aAPCs in LNs. Because DCs were co-cultured with tumor cell lysate, MHC molecules displayed a diversity of tumor antigens, effectively activating polyclonal T lymphocytes. Furthermore, imiquimod polarized macrophages to M1 macrophages after phagocytosing aAPCs, increasing tumor-killing capacity. This improved approach is more versatile and has demonstrated promising therapeutic effects in solid tumors.

Unlike aAPCs, biomimetic cancer vaccines stimulate adaptive immune responses by activating APCs in LNs. However, due to the presence of efferent lymphatic arteries, vaccines quickly move out of LNs, reducing their uptake by APCs. Furthermore, APC-presented antigens are always presented and processed alongside MHC II, implying that CD8^+^ T cell-induced cellular immunity is not activated.^203,204^ To overcome the problems, Xie et al. created a tumor cell membrane-coated biomimetic cancer vaccine (A/M/C-MNC).^205^ CpG-ODN, a Toll-like receptor (TLR) agonist, was adsorbed on the core of Fe_3_O_4_ MNCs (C-MNCs). N_3_-labeled cancer cell membranes were then coated on C-MNCs and bound to DBCO-modified anti-CD205 via bioorthogonal reaction. CD8^+^ DCs recognized and took up A/M/C-MNC preferentially in the presence of CD205, enhancing MHC I cross-presentation and CD8^+^ T cell activation. Moreover, A/M/C-MNC increased CD8^+^ T cell proliferation and IFN-γ secretion. A/M/C-MNC demonstrated substantial anticancer activity against five different tumor models, indicating considerable promise for using this innovative platform for cancer therapy.

In addition to stimulating T-cell immune responses, modulation of TME can also enhance tumor immunotherapy. Researches show that an increase of intratumoral H_2_O_2_ promotes the Fenton reaction, which leads to ferroptosis of tumor cells and releases tumor antigens.^206,207^ Inspired by this, Xie et al. created a biomimetic system (Pa-M/Ti-NC) that reduced T cell immunosuppression while triggering ferroptosis.^208^ TGF-β inhibitor (Ti) was loaded onto the N_3_-labeled leukocyte membranes. The membranes were then modified with DBCO-PD-1 antibody (Pa) and were used to encapsulate magnetic nanoparticles. The nanoparticles infiltrated into tumor tissue due to the magnetic effect. Pa linked to PD-L1 on T cells to alleviate T cell immunosuppression. Ti is linked to TGF-β, reducing T_reg_ levels while increasing antigen-presenting of APCs. Simultaneously, the H_2_O_2_ levels in TME increased. The increasing H_2_O_2_ facilitated the Fenton reaction which generated ROS. ROS induced ferroptosis in tumor cells and the release of tumor antigens, increasing the immunogenicity of the tumor. The results demonstrated that Pa-M/Ti-NC suppressed tumor growth with good anti-metastatic effects. This combination of immunological regulation and ferroptosis increases immune cell activity while enhancing tumor tissue immunogenicity, making it available for tumor immunotherapy.

Exosomes are membrane-bound nanoscale vesicular particles produced by cells. Because of their similarities, exosomes can mimic the function of their originating cells in cancer therapy.^209,210^ However, the clinical therapeutic effectiveness of exosomes is typically poor. Although genetic modification and chemical cross-linking are used to modify exosomes with specific antibodies or immunostimulatory/inhibitory molecules, these methods are complicated and time-consuming. To overcome these challenges, Xie et al. designed a bioorthogonal reaction-based pH-responsive exosome (M1 Exo-Ab) to improve tumor immunotherapy.^211^ DBCO-modified anti-CD47 antibody (aCD47) and anti-signal regulatory protein alpha (SIRP) antibody (aSIRP) were bonded to M1 macrophage exosomes (M1 Exos) and a pH-sensitive benzoic imine bond. M1 Exo-Abs accumulated in tumor cells overexpressing CD47 due to aCD47. Low pH at the tumor site broke the benzoic imine bond of M1 Exo-Ab, releasing aCD47 and aSIRP, which then inhibited the receptors SIRP on macrophages and CD47 on tumor cells, eradicating the “don’t eat me” signal and improving macrophage phagocytic activity. Meanwhile, M1 Exo could reprogram M2 macrophages into M1 macrophages, increasing the anti-cancer efficacy of M1 Exo-Ab. This bioorthogonal exosome modification strategy can be employed in different exosome systems, making it a broad exosome engineering technique.

In a similar study, Jiang et al. developed a neutrophil-derived exosome functionalized with sub-5nm ultra-small Prussian blue nanoparticles (uPB-Exos) by bioorthogonal reaction for the treatment of rheumatoid arthritis (RA).^212^ Due to neutrophil chemotaxis to inflammatory sites,^213–217^ uPB-Exos accumulated in activated fibroblast-like synoviocytes, where they neutralized pro-inflammatory chemicals and reduced inflammatory stress. In addition, uPB-Exos targeted inflammatory synovitis and entered deep into the cartilage. Using MRI, RA may be accurately diagnosed in vivo with high sensitivity and specificity. When compared to anti-inflammatory drugs and biological antibodies, uPB-Exos have higher targeting capabilities, which can improve therapeutic efficacy while reducing unnecessary side effects. Most importantly, uPB-Exos respond to increasing inflammatory molecules in advanced RA, breaking the barrier that no available medicines have a detectable therapeutic effect on advanced RA.^218^ As a result, these bioorthogonal chemistry-based exosomes have a high potential for clinical use in the diagnosis and treatment of RA.

### Constructing cell-based DDSs by bioorthogonal chemistry

Natural targeting, inflammatory chemotaxis, and immunological control are actions performed by distinct types of cells. The use of bioorthogonal reactions to combine drug-loaded nanoparticles, antibodies, or therapeutic platelets with specific cells exploits the innate targeting capacity of cells to deliver drugs or increase the immunological regulatory function of cells, which is regarded as a successful technique for developing cell-based DDSs (Fig. 6b).

#### Conjugating nanoparticles to the cell membrane

One concept is to conjugate nanoparticles on the surface of living cells to transport therapeutic agents and improve their efficacy, known as the “backpack” strategy.^219–222^ This approach produced positive results in preclinical studies. However, mechanisms for attaching nanoparticles to cell surfaces such as electrostatic adsorption, hydrophobic interactions, and antigen-antibody interactions are limited to specific cell types. Furthermore, these methods have the potential to interfere with cell function (such as cell signaling). Bioorthogonal chemistry is a low-toxicity method for attaching nanoparticles to cell surfaces without interfering with biomolecule function. Several advances in bioorthogonal chemistry have been made in the coupling of nanoparticles to cell membranes.

CAR-T therapy has remarkable success in blood cancer, but due to the complex TME of solid tumors, it has not been efficiently implemented in the treatment of solid tumors.^223,224^ Cai et al. developed a nanophotosensitizer-engineered CAR-T (CT-INPs) to improve therapeutic efficacy.^225^ Indocyanine green nanoparticles (INPs) attached to CAR-T cells through a bioorthogonal reaction. When exposed to NIR laser irradiation, CT-INPs induced a minor photothermal reaction. These photothermal interventions boosted CT-INP infiltration and recruitment by disrupting the extracellular matrix, dilating blood vessels, relaxing dense tissue, and increasing chemokine release, all of which improved CAR-T immunotherapy efficiency. In a similar work, Cai et al. created an intelligent IL-12 nano-engineered CAR-T cell (INS-CAR-T) using a bioorthogonal reaction to deliver IL-12 nanoparticles to tumor tissues.^226^ The released IL-12 increased the secretion of anticancer cytokines and tumor chemokines, activated CD8^+^ CAR-T cells in tumors, and resulted in efficient antitumor immunotherapy.

In addition to boosting CRA-T treatment against solid tumors by regulating TME, interfering with the cholesterol metabolism of T cells can also improve the tumor-killing capacity. Zhang et al. used a bioorthogonal reaction to bind liposomes containing cholesterol esterase inhibitor Avasimibe (Ava) to the surface of T cells, enhancing the tumor-killing ability of T cells.^108^ High cholesterol concentrations in T cell membranes have been shown to trigger TCR aggregation and immunological synapse formation, boosting T cell tumor-killing ability.^227^ The authors first exploited the lipid insertion concept to introduce Tre-modified phospholipids into the surface of T cells. Then, using bioorthogonal reactions, BCN-modified Ava-loaded liposomes were bound to the T cell membrane. Liposomes stably persisted on the cell surface, blocking T cell cholesterol esterase activity by slowly releasing Ava and increasing cholesterol levels in T cell membranes. Elevated cholesterol levels induced TCR aggregation and prolonged T cell activation, both of which contributed to efficient tumor killing. Based on bioorthogonal chemistry, this strategy combining adoptive cell therapy with metabolic therapy can boost T cells’ tumor-killing capabilities against mouse melanoma and glioblastoma. In addition to T cells, researchers have also bound Cy5.5-loaded BCN CNPs (BCN-CNPs-Cy5.5) or DBCO-modified paclitaxel PLGA nanoparticles to mesenchymal stem cells (MSCs) via bioorthogonal reactions.^228,229^ MSCs can identify inflammatory signals generated by neutrophils and move to the tumor inflammatory milieu, enabling tumor monitoring imaging and chemotherapeutic targeting.

In addition to the direct use of bioorthogonal chemistry to attach nanoparticles to cell membranes, it can also be used to increase the targeting delivery of cell “backpacks”. Natural killer (NK) cells can kill tumor cells without sensitizing them to antigens and secreting cytokines.^230^ However, the poor tumor homing ability and the downregulated tumor killing in the immunosuppressive TME prevent the use of NK cells in solid tumor therapy.^231^ Given this, Cai et al. constructed engineered NK cells (N_3_-NK-NPs) for immunotherapy of solid tumors.^232^ The bioorthogonal groups (N_3_ and BCN) were utilized to label NK and tumor cells, respectively. Redox-responsive IL-21 nanoparticles (ILNPs) modified with anti-CD45 were then constructed, and they were coupled to NK cells by antigen-specific reactions. As artificial targeting receptors/ligands, N_3_ and BCN achieved efficient recognition between NK and tumor cells which further promoted NK cell infiltration into tumor tissue. Meanwhile, the sustained release of IL-21 promoted the proliferation, activation, and persistence of NK cells, which was somehow similar to “cellular autocrine”. This bioorthogonal chemistry-based “backpack” system provides a reliable strategy for NK cell therapy with high and safe therapeutic efficacy.

As a result, bioorthogonal chemistry is an excellent tool for the cellular “backpack” strategy, which can maintain the normal physiological functioning of cells while also enhancing therapeutic efficacy. In addition to T cells and MSCs, the bioorthogonal reactions of drug-loaded nanoparticles on the membrane of other types of cells such as macrophages, DC cells, and B cells may broaden the application of the cell “backpack” strategy in disease treatment.

#### Conjugating biomolecules to the cell membrane

Modification of biomolecules such as antibodies on cell membranes is predicted to enhance cell therapy efficacy. However, genetic engineering is inapplicable to highly differentiated cells. NK cells, for example, have difficulty digesting endogenous genetic material due to their unique features, resulting in limited transgenic expression.^233^ Additionally, genetic engineering may affect the physiological function of cells with costly and time-consuming procedures. Bioorthogonal chemistry can rapidly conjugate biomolecules onto cell membranes, effectively circumventing the constraints of genetic engineering. For example, Huang et al. coupled CD22 ligands onto NK cells to generate modified NK cells with tumor-targeting capabilities.^234^ These NK cells displayed high selectivity and cytotoxicity to CD22^+^ lymphoma cells in a CD22-dependent manner, which had a considerable protective effect in tumor-bearing mice. Also, Yuan et al. designed an engineered CAR-T cell modified with hyaluronidase (HAase) and checkpoint blocking antibody aPDL1 via bioorthogonal chemistry to improve therapeutic efficacy against solid tumors.^235^ HAase degraded hyaluronan and disrupted the tumor extracellular matrix, allowing CAR-T cells to penetrate deeply into solid tumors. In addition, modification of aPDL1 improved the anti-tumor ability of CAR-T. In this work, CAR-T cells were modified to overcome the poor tumor penetration, while the engineered modified aPDL1 played a synergistic role with CAR-T to enhance the therapeutic effect on solid tumors without affecting the normal function of CAR-T. This engineering strategy of “overcoming weaknesses and enhancing advantages” can be extended to other adoptive cell therapy and has great potential for clinical application.

Additionally, Wang et al. created an immune checkpoint ligand-engineered β cell to treat type 1 diabetes mellitus (TIDM) by suppressing islet-specific T cell function.^236^ It has been observed that β cells are deficient in PD-L1 (a co-inhibitory ligand for PD-1), CD86 (a co-inhibitory ligand for CTLA-4), and Gal-9 (a co-inhibitory ligand for TIM3), all of which linked to the development of T1DM.^237–239^ Based on this, the researchers employed DBCO dendrimers to attach PD-L1, CD86, and Gal-9 to N_3_-labeled β cells, creating PD-L1/CD86/Gal-9-modified β cells. They used pancreatic extracellular matrix (PAN-ECM) scaffolds to implant β cells. Within PAN-ECM, modified β cells restored the immunogenic islet milieu and drove islet antigen-specific T cell depletion by binding to autoreactive T cells, reversing premature hyperglycemia. When compared to platelets with high PD-L1 expression, these modified β cells can trigger islet-specific antigen T depletion without damaging normal tissues, which can alleviate or prevent diabetes progression.

Recent research has demonstrated that changes in the physical features of antibodies, such as aggregation propensity, might serve as the basis for initiating specific functions such as cytotoxicity.^240^ Urano et al. exploited a bioorthogonal reaction on the cell membrane to promote antibody aggregation and thereby enhanced cell signaling pathways.^241^ They first coupled Tz, MTz, and BCN with PEG before binding them to the protein labeling site (N-hydroxyhydroaminovinyl ester) to create a collection of antibody clickers (ABC). By raising the effective concentration of antibody that binds to the target antigen, bioorthogonal interaction between cell surface antibodies can be enhanced. When trastuzumab was given to HER2-expressing SKBR3 cell lines, the trastuzumab aggregated due to a bioorthogonal reaction. However, instead of inhibiting cell growth, the aggregation promoted it. Immunostaining revealed a significant nuclear phosphorylated ERK signal, implying that antibody aggregation triggered cross-phosphorylation. This study expands the options for cell-based therapy. On the one hand, the unique activities produced by antibody aggregation may improve illness treatment outcomes; on the other hand, such bioorthogonal reaction-based antibody aggregation can be used as a synthetic biology tool to modify intracellular signaling pathways of interest.

#### Conjugating functionalized platelets to the cell membrane

Although conjugating nanoparticles on the cell surface can allow targeted delivery of nano-drugs, the nanomaterials currently used in the cellular “backpacks” strategy are liposomes or polymers with basic structures which have no sensitivity to low pH or enzyme in tumor tissue, making accurate and regulated drug release at tumor location problematic.

The adaptability of bioorthogonal reactions helps the development of cell combination strategies in which one cell serves as a delivery vehicle and the other as a drug carrier. Gu et al. created an HSC-platelet combination preparation for the treatment of acute myeloid leukemia (AML) by combining PD-1-modified platelets with hematopoietic stem cells (HSCs).^242^ To establish cell-cell conjugation, N_3_-modified HSCs were mixed with DBCO-modified platelets in a 1:1 ratio. The cell-cell conjugation could travel to the bone marrow after intravenous injection due to the action of HSC. The platelets were then stimulated to form several platelet-derived vesicles, boosting the local release of PD-1. This cell-cell conjugation effectively suppressed AML by stimulating T cells, resulting in an efficient immune response. This cell combination technique provides superior biocompatibility and bioreactivity because the cells are entirely derived from the host. Among them, the bioorthogonal reaction is one of the most significant components in the successful development of cell-cell conjugation, because researchers can purposely change the ratio of the two types of cells to achieve the best cell survival and therapeutic effect. This bioorthogonal chemistry-based cell combination technique will be applied to other types of cells in the future, and it is envisaged that a range of cell-cell conjugations with varied functionalities will be built for the treatment of various diseases.

### Constructing bacteria-based DDSs by bioorthogonal chemistry

Bacteria-based DDSs bring ingenious synergies to various biomedical applications for interdisciplinary research.^243^ Bacteria autonomously sense and respond to dynamic physiological signals and prevent or exacerbate disease progression by preferentially migrating to disease regions through self-propulsion. Coupling nanoparticles to bacterial surfaces via bioorthogonal chemistry allows efficient delivery of drugs to disease sites with higher specificity and lower systemic toxicity to kill disease-causing microorganisms than conventional therapeutic approaches, offering great potential on bacterial-based DDSs for clinical applications.

Considering that *E. coli* has a strong resistance to lead and the ability to detoxify from certain concentrations of lead, Zhang et al. reported an integrated bio/non-biological hybrid bioreactor to integrate lead detoxification and ROS elimination and further achieve lead damage mitigation.^244^ Briefly, a non-pathogenic bacterium, *Escherichia coli* MG1655 (Bac), was bioorthological modified with antioxidant cerium oxide nanoparticles (Ceria), a versatile antioxidant reagent with good antioxidant activity at physiological pH on its surface. Bac could actively carry Ceria and accumulated in organs with high lead concentrations. Depending on the resistance of bacteria to lead, excess toxic metals are mainly bio-absorbed and the imbalance of oxidative stress at pathological sites is simultaneously repaired by Ceria. In vitro and in vivo results indicated that this hybrid bioreactor was safe for biomedical applications and showed significant lesion targeting, lead elimination and antioxidant efficacy.

In addition, Jiang et al. designed a novel intelligent and biocompatible theranostic strategy based on bacteria-induced gold nanoparticle (GNP) aggregation for bacterial surface-enhanced Raman scattering (SERS) imaging and antimicrobial PTT therapy.^245^ TCO derivatives of vancomycin (Van-TCO) were synthesized and bound to the bacterial surface by forming five hydrogen bonds with the C-terminal d-Ala-d-Ala motif of peptidoglycan on the cell wall of G^+^ bacteria. Next, Tz-modified gold nanoparticles (GNP-Tz) aggregated on the bacterial surface through a bioorthogonal reaction. Plasmonic excitonic coupling effects between adjacent GNPs triggered strong electromagnetic fields and high NIR absorption, which enabled SERS imaging and photothermal ablation of bacterial pathogens without causing damage to healthy tissue. This method based on bacteria-induced bioorthogonal GNP aggregation showed great potential for application in bacterial SERS imaging and antimicrobial PTT studies.

### Constructing phage-based DDSs by bioorthogonal chemistry

Phages are viruses that infect bacteria and are common in the gastrointestinal system. They target specific harmful bacteria without disrupting normal microbiota. Therefore, phages can be employed as perfect drug delivery vehicles to deliver drugs to specific tissues or organs where the target bacteria are present.^246–248^

In light of this, Zheng et al. created a bioorthogonal phage-based gut microbiome manipulation method for the treatment of chronic colon cancer (CRC).^249^ First, the researchers found a phage that particularly cleaved *F. nucleatum* in saliva since there was a substantial enrichment of *F. nucleatum* in the feces of CRC patients. To generate N_3_-labeled phages, L-azidoalanine was added to the phage culture medium with nuclear *F. nucleatum*, and N_3_ was bound to the phage’s outer capsid via the protein shell production mechanism. They next synthesized DBCO-modified irinotecan (IRT)-loaded dextran nanoparticles (D-IDNPs) and attached them to the phage surface using bioorthogonal reactions. This system contained two functional sections. One such agent was a phage, which could target and eliminate nuclear *F. nucleatum* in tumors. The other was nanoparticle enriched in CRC, increasing the efficacy of chemotherapy drugs. Additionally, the glucan shells of D-IDNPs were also shown to enhance the growth of endogenous Clostridium butyricum which increased colonic short-chain fatty acid (SCFA) levels. This strategy offers a novel approach to the treatment of CRC, as well as a model for the treatment of other cancers. If bacteria in tumors can be identified, phage-based DDSs based on bioorthogonal reactions can be produced for cancer therapy. This will be a strategy with low toxicity, great efficiency, and low cost.

## Application of bioorthogonal chemistry in developing multi-functional hydrogel

Hydrogel is a hydrophilic three-dimensional network structure. Due to their viscoelasticity and high-water content, hydrogels are considered potential biological scaffolds that mimic the natural extracellular matrix (ECM) for local drug or therapeutic cell delivery.^250–252^ Currently, many cross-linking methods have been used to prepare covalently cross-linked hydrogel networks. For example, cross-linking peptide hydrogels via the horseradish/hydrogen peroxidase (HRP/H_2_O_2_) system have been developed.^253^ However, these cross-linking systems with small molecule chemical cross-linkers may be potentially toxic to the encapsulated cells or the body tissues. Bioorthogonal reactions, due to their rapidity, efficiency, high selectivity, and good biocompatibility in vivo and in vitro have emerged as an emerging cross-linking strategy for constructing hydrogels. Recent advances in bioorthogonal chemistry in the construction of hydrogel systems are described below.

### Constructing hydrogel scaffolds

As an efficient and low-toxic cross-linking strategy, bioorthogonal reactions are gradually being applied to the construction of hydrogel scaffolds. David et al. proposed a bioorthogonal cross-linked hyaluronate-collagen hydrogel for in situ filling of corneal defects without any sutures, initiators, or catalysts.^254^ They mixed dialyzed N_3_-modified hyaluronate (HA-azide) with DBCO-modified collagen (Col-DBCO) to produce hyaluronic acid-collagen hydrogels via the SPAAC reaction under biological conditions. Compared to collagen hydrogels, this hydrogel is stronger and more closely resembles the natural corneal matrix, offering excellent potential as a biomaterial for corneal repair and regeneration. Chen et al. prepared PLG hydrogels by mixing N_3_ with DBCO-modified poly (L-glutamic acid) (PLG) under physiological conditions by bioorthogonal reactions.^255^ Bone marrow mesenchymal stem cells (BMSCs) were able to maintain a high survival rate when cultured inside the hydrogel in vitro. Subsequent simultaneous bonding of cell adhesion peptide c(RGDfK) and neurocalmodulin mimetic peptide (N-cad) to the hydrogel network significantly promoted the adhesion of BMSCs on the hydrogel surface and the proliferation of chondrocytes inside the hydrogel. On the other hand, the introduction of N-cad significantly increased the production of cartilage-specific matrix and the expression of chondrogenesis-related genes and proteins in the hydrogel. This study proposes a new approach for the construction of multiple biofunctionalized hydrogel platforms and is expected to be applied to the in vitro preservation and local delivery of immune cells with clinical translation prospects.

In addition, the combination of a bioorthogonal cross-linker and a stimulus-responsive cross-linker for the construction of smart responsive hydrogels can achieve sustained drug release and synergistic effects of multiple therapeutic strategies. Kim et al. designed a quadratically coupled hybrid gel (M-NO) for nitric oxide scavenging and sequential drug release.^256^ Solutions of the individual components of M-NO were injected into the joint via a dual injection system and rapidly gelated in situ by bioorthogonal ligation reaction. The bis-alkyne-modified NO-responsive cross-linker, DA-NOCCL, is incorporated into the N_3_-functionalized hyaluronic acid backbone. Under the stimulation of NO, DA-NOCCL rapidly breaks, thereby disrupting the gel structure and thus releasing the encapsulated drug, which effectively relieves or even eliminates arthritic symptoms in a rheumatoid arthritis model.

### Coupling treatment components

In addition to the use of bioorthogonal chemistry to construct hydrogel scaffolds, bioorthogonal chemistry can also be used to attach therapeutic agents to the hydrogel backbone to achieve long-term retention of therapeutic agents in the lesion. Ren et al. achieved long-term in vivo EV retention by selectively immobilizing N_3_-modified EV (Az-EV) in DBCO-modified collagen hydrogels via bioorthogonal chemistry.^257^ MSC-derived Az-EV triggered more robust host cell infiltration, angiogenesis, and immunomodulatory responses when immobilized in collagen hydrogels by bioorthogonal chemistry at one-tenth the dose required for non-immobilized EV. This technique is expected to be applied to CAR-T and others to improve their intratumoral retention time and improve the efficacy of cell therapy.

## Conclusion and outlook

**Fig. 7:**
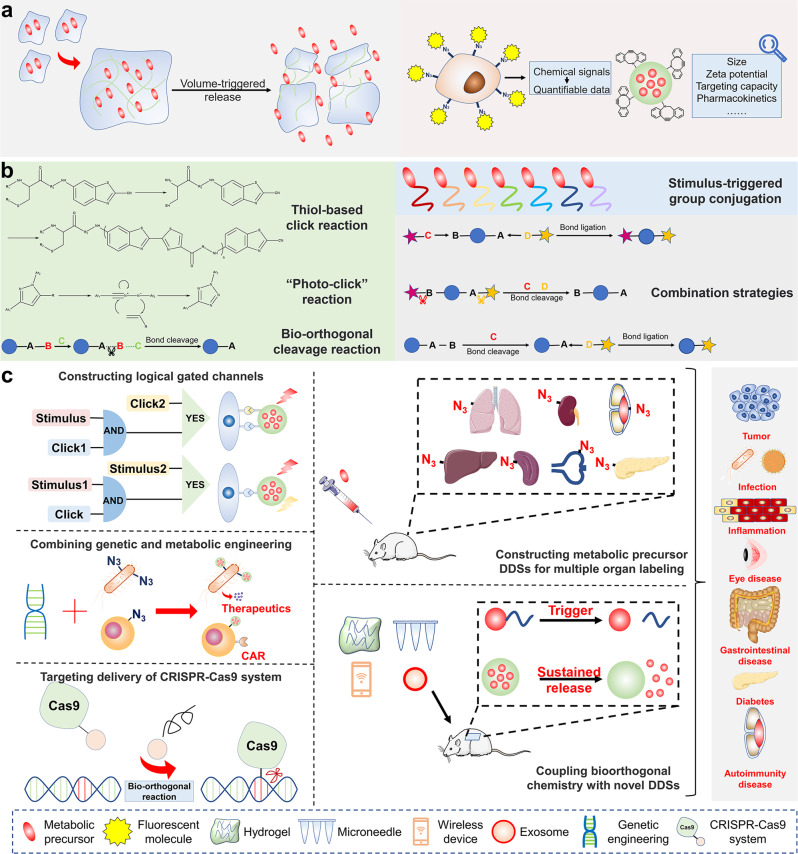
Outlook of bioorthogonal chemistry in developing DSSs. **a** Precise regulation of bioorthogonal metabolic labeling. 1. Sustained release of metabolic precursors by constructing a volume-triggered release system, resulting in a stable bioorthogonal label in vivo. 2. Developing fluorescent markers for accurate quantification of cell surface bioorthogonal groups while comprehensively assessing the physicochemical and pharmacokinetic properties of paired group-modified nanocarriers. **b** Enriching the bioorthogonal toolkit. Many intelligent metabolic precursors will be created by modifying stimuli-triggered groups. Developing various novel bioorthogonal reactions or combining multiple bioorthogonal reactions will improve the selectivity and functionality of DDSs. **c** Cross-integration with emerging technologies. 1. Constructing logical gated channels for precise targeting and release of drugs. 2. Combining genetic and metabolic engineering to develop multi-functional cell-based DDSs. 3. Targeting delivery of CRISPR-Cas9 system using bioorthogonal chemistry. 4. Constructing metabolic precursor DDSs that can selectively label different organs or tissues for the treatment of various diseases. 5. Coupling bioorthogonal chemistry with novel DDSs such as hydrogels, microneedles, wireless devices, and exosomes to achieve stimulus-triggered and sustained drug release

In this review, we discussed recent advances in developing active targeting and multi-functional DDSs via bioorthogonal chemistry. Bioorthogonal chemistry is mild with great efficiency, selectivity, and low toxicity, and does not affect normal biological processes.^258–260^ Bioorthogonal groups (such as N_3_) can be labeled onto tumor tissues via metabolic engineering. By modifying paired groups (such as DBCO) on nanoparticles, the nonspecific distribution, restricted tumor targeting, and low penetration of nanomedicines can be overcome. Furthermore, antibodies, cytokines, vaccinations, and viral vectors can selectively target immune cells such as DCs and T cells via bioorthogonal chemistry to stimulate powerful immune responses. This method is also applicable to the targeted delivery of antibacterial drugs to pathogenic microorganisms. In addition to the targeted delivery, nanoparticles, antibodies, and platelets can connect to the cell surface via bioorthogonal reactions, creating multi-functional bio-inspired DDSs. On the one hand, bioorthogonal chemistry can be used to construct biomimetic DDSs with superior targeting or immunomodulatory capabilities. On the other hand, bioorthogonal chemistry enables the anchoring of diverse biomolecules or nanoparticles on the cell surface, creating multi-functional cell-based, bacteria-based and phage-based DDSs. In addition, the construction of hydrogels using bioorthogonal chemistry has also been used for the treatment of diseases. Despite significant advances in developing DDSs, several challenges of bioorthogonal chemistry remain to be overcome (Fig. 7).

### Precise regulation of bioorthogonal metabolic labeling

Precise modulation of bioorthogonal metabolic labeling is the key to its application in disease treatment. First, improving the tissue selectivity of metabolic percursors and reducing off-target effects are necessary. Selective targeting of tumors can be achieved using stimuli-responsive unnatural sugars. However, due to the weak difference between tumor microenvironment and normal tissue, the uptake of non-natural sugars by normal cells cannot be completely blocked. To obtain unnatural sugars with intrinsic cancer marker selectivity, the design of excitation devices with tumor-specific reactivation of cellular metabolic pathways is particularly important. Currently, tumor overexpression enzymes, including cathepsin B, HDAC, and CTSL, are mainly used to develop non-natural glycans that can be converted from dormant to activated state only in tumor tissues.^87,98^ Considering that many of these enzymes are also present in normal cells, it may be necessary to design co-excitation devices using multiple enzymes simultaneously to improve tumor selectivity. Due to tumor heterogeneity, a comprehensive evaluation and screening of metabolic precursors with optimal tumor selectivity and labeling efficiency are required. In addition, the use of stimuli-responsive DDSs for the delivery of metabolic precursors can improve labeling efficiency and prolong the circulation time of metabolic precursors to a certain extent.^260–263^ Second, the density, stability, and uniformity of bioorthogonal groups on cell membranes should be evaluated. As glycans, proteins, and phosphatidylcholines are predominantly found on the outer surface of cell membranes, bioorthogonal groups are mostly found there. However, due to the randomization of the orientation inside and outside the membrane, it is impossible to verify that all bioorthogonal groups on the surface of membrane-coated nanoparticles are outside.^76^ Current fluorescent probes, such as DBCO-Cy5, cannot react with N_3_ on the cell membrane in a 1:1 ratio due to spatial site resistance, making it difficult to accurately quantify the metabolic label on the cell membrane. Furthermore, because of the exchange of substances between cells and the extracellular environment, as well as the rapid metabolism of glycans, the density of these bioorthogonal groups is constantly changing. This may result in the loss of modified nanoparticles or biomolecules on cell membranes. For in vitro research, cultivating cells in a medium containing bioorthogonal metabolic precursors is an effective strategy for maintaining the density of bioorthogonal groups. However, for in vivo studies, large amounts of metabolic precursors need to be injected to maintain stable metabolic labels on the surface of target cells, causing potential damage to normal tissues. Therefore, future research will break through from the following two demands: 1) developing bio-activated fluorescent probes with small spatial site resistance to achieve accurate quantification of bioorthogonal groups on the cell surface; 2) developing in situ metabolic precursor reservoirs, which can only release metabolic precursors when the system is activated under specific conditions to achieve continuous and stable metabolic labeling in vivo. Third, the alterations in characteristics and effective concentrations of nanocarriers generated by the bioorthogonal modification necessitate further evaluation. DBCO may change the size, and potential of nanoparticles, and the density of DBCO may affects the targeting and pharmacokinetic properties of nanomedicines. In addition, because of their small size, N_3_ will not be depleted by DBCO in a single dose, so multiple dosing is required, and the frequency and the dose, may vary depending on the density of DBCO on the nanocarrier surface. Therefore, a comprehensive characterization analysis of nanocarriers is needed to determine the optimal ratio of bioorthogonal groups to nanocarriers for the best therapeutic effect. Also, means to realize the spatio-temporal control of bioorthogonal reactions by direct or indirect methods for their biosafety assessment need to be developed.

### Enriching the toolkit

Considering that bioorthogonal reactions have been increasingly used in DDSs, the diverse bioorthogonal toolkit needs to be expanded to develop smart DDSs. To create different bioorthogonal reactions to make the toolkit practically useful and valuable, we should first pay attention to the bioorthogonality of the tool and the by-products, which must both be stable and biocompatible and must be non-biologically active. In addition, the reaction pairs should also be abiotic and undergo rapid, potent interactions in the cellular environment.^77^ This process can be simply achieved in the future with the cross-fertilization of artificial intelligence (AI) and organic chemistry. Furthermore, considering that the selectivity of SPAAC is compromised by cyclooctyne and thiol side reactions on the surface of biomolecules and iEDDA is limited due to TCO’s cis-trans isomerism,^58^ different forms of click reactions have been developed. For example, the “photo-click” reaction between an alkene and a nitrile imine provides a unique bioorthogonal technique due to its well-controlled spatial and temporal features.^264,265^ In cell engineering, thiol and maleimide click reactions are also extensively used.^266^ In addition to the bioorthogonal ligation reactions highlighted in this review, bioorthogonal cleavage reactions (also termed bioorthogonal-triggered release) have emerged as a new type of bioorthogonal reaction for DDS development.^267,268^ Bioorthogonal cleavage reactions are deprotection reactions triggered mainly by small molecules or enzymes. The applications in recent years are mainly focused on protein activation and prodrug activation. Considering the high specificity of the reaction, the trigger can be delivered or localized to the disease site, thus triggering the release of the therapeutic drug from the DDS at the lesion. For example, Gu et al. loaded a Pd catalyst in a microneedle patch and implanted the triggering agent into a subcutaneous tumor via microneedle, thus achieving local prodrug activation at the tumor site.^269^ In addition, the use of bioorthogonal cleavage reactions to construct biological scaffolds capable of depolymerization under specific conditions would provide a novel strategy for targeted delivery and sustained release of therapeutic cells or macromolecular drugs. Finally, to further improve the selectivity of bioorthogonal reactions, two or more bioorthogonal reactions can be combined and cooperated. Such strategies include, but are not limited to, two bioorthogonal linkage reactions, two bioorthogonal cleavage reactions, and the combination of a bioorthogonal linkage reaction and a bioorthogonal cleavage reaction. By mixing multiple bioorthogonal reactions in one system and controlling two different sites simultaneously or sequentially, this combination strategy has been used for the precise modification of peptides and proteins.^270^ It is foreseen that if this “dual bioorthogonal” system is applied to the construction of DDSs, the targeting capacity of DDSs will be greatly improved.

### Cross-integration with emerging technologies

Although bioorthogonal chemistry is gradually being applied to the development of DDSs, most research has focused on improving the tumor targeting of therapeutics and the construction of biomimetic DDSs. This is mainly limited by the development of other technologies in drug delivery. With the rapid development of nanotechnology, materials science, and biomedicine, bioorthogonal chemistry is expected to be applied to more emerging fields, providing a constant potential for the construction of intelligent multi-functional DDSs. For example, the use of bioorthogonal chemistry and stimulus-triggered motifs to construct logically gated channels allows for precise targeting and release of drugs. The combined use of bioorthogonal chemistry and synthetic biology allows for the construction of engineered cells^235^ or microalgae^271^ with multi-functionalities. This engineering approach combines the advantages of genetic engineering and metabolic engineering without interfering with each other, greatly enriching and enhancing cellular functions and providing new ideas for the development of efficient periplasmic cell therapies. Also, combining bioorthogonal chemistry with novel DDSs such as hydrogels, microneedles, wireless devices, and exosomes is expected to achieve controlled release of drugs. Moreover, we should not limit the treatment of tumors. As described in the article, the use of unnatural sugars can effectively label tumor tissues and achieve targeting of DDSs to tumor tissues. If we can develop bioorthogonal metabolic precursors that label different organs to enable the targeting of therapeutic agents to different organs, this will greatly expand the use of bioorthogonal-based DDSs in the treatment of autoimmune diseases, bacterial infections, inflammation, and neurological diseases.^272^ Furthermore, the use of bioorthogonal cleavage reactions is also expected to overcome the systemic toxicity arising from off-target gene editing,^273^ while providing a proven strategy for in vivo editing and modulation of immune cells.

Precise delivery and intelligent drug release are two essential conditions for a DDS to be therapeutically effective. As we strive to apply bioorthogonal chemistry to DDSs, more and more selectively targeting and multi-functional DDSs will be developed. Here, we highlight the latest developments and applications of bioorthogonal linkage reactions in DDSs, while providing insights into some of the emerging bioorthogonal cleavage reactions in DDSs and summarizing the future challenges of this exciting tool. It is believed that efficient, low-toxic, and promising bioorthogonal chemistry has a broad application in the future research for developing smart DDSs under the background of multidisciplinary cross-fertilization.

## References

[1] Zhao Z, Ukidve A, Kim J, Mitragotri S (2020). Targeting strategies for tissue-specific drug delivery. Cell.

[2] Park H, Otte A, Park K (2022). Evolution of drug delivery systems: From 1950 to 2020 and beyond. J. Control. Rel..

[3] Hu QQ, Li H, Wang LH, Gu HZ, Fan CH (2019). DNA nanotechnology-enabled drug delivery systems. Chem. Rev..

[4] Becker ML, Burdick JA (2021). Introduction: polymeric biomaterials. Chem. Rev..

[5] Elsabahy M, Wooley KL (2012). Design of polymeric nanoparticles for biomedical delivery applications. Chem. Soc. Rev..

[6] Al-Jamal WT, Kostarelos K (2011). Liposomes: from a clinically established drug delivery system to a nanoparticle platform for theranostic nanomedicine. Acc. Chem. Res..

[7] Zheng QY (2021). The recent progress on metal-organic frameworks for phototherapy. Chem. Soc. Rev..

[8] Ma, Z. H., Mohapatra, J., Wei, K. C., Liu, J. P. & Sun, S. H. Magnetic nanoparticles: Synthesis, anisotropy, and applications. *Chem. Rev*. 10.1021/acs.chemrev.1c00860 (2021). 10.1021/acs.chemrev.1c00860 34968046

[9] Bieniek A (2021). MOF materials as therapeutic agents, drug carriers, imaging agents and biosensors in cancer biomedicine: Recent advances and perspectives. Prog. Mater. Sci..

[10] Li ZT (2021). Cell-based delivery systems: emerging carriers for immunotherapy. Adv. Funct. Mater..

[11] Chen ZW, Wang ZJ, Gu Z (2019). Bioinspired and biomimetic nanomedicines. Acc. Chem. Res..

[12] Chen ZW, Hu QY, Gu Z (2018). Leveraging engineering of cells for drug delivery. Acc. Chem. Res..

[13] Zhai YH (2017). Preparation and application of cell membrane-camouflaged nanoparticles for cancer therapy. Theranostics.

[14] Shi JJ, Kantoff PW, Wooster R, Farokhzad OC (2017). Cancer nanomedicine: progress, challenges and opportunities. Nat. Rev. Cancer.

[15] Fang RH, Jiang Y, Fang JC, Zhang LF (2017). Cell membrane-derived nanomaterials for biomedical applications. Biomaterials.

[16] Vargason AM, Anselmo AC, Mitragotri S (2021). The evolution of commercial drug delivery technologies. Nat. Biomed. Eng..

[17] Zhou L, Zhang P, Wang H, Wang D, Li Y (2020). Smart nanosized drug delivery systems inducing immunogenic cell death for combination with cancer immunotherapy. Acc. Chem. Res..

[18] Zhang Y, Xu C, Yang XL, Pu KY (2020). Photoactivatable protherapeutic nanomedicine for cancer. Adv. Mater..

[19] Bertrand N, Wu J, Xu XY, Kamaly N, Farokhzad OC (2014). Cancer nanotechnology: the impact of passive and active targeting in the era of modern cancer biology. Adv. Drug Deliv. Rev..

[20] Maeda H, Nakamura H, Fang J (2013). The EPR effect for macromolecular drug delivery to solid tumors: Improvement of tumor uptake, lowering of systemic toxicity, and distinct tumor imaging in vivo. Adv. Drug Deliv. Rev..

[21] Xie Y (2020). Stromal modulation and treatment of metastatic pancreatic cancer with local intraperitoneal triple mirna/sirna nanotherapy. ACS Nano.

[22] He YC (2020). Sequential intra-intercellular delivery of nanomedicine for deep drug-resistant solid tumor penetration. ACS Appl. Mater. Interfaces.

[23] Cabral H (2011). Accumulation of sub-100 nm polymeric micelles in poorly permeable tumours depends on size. Nat. Nanotechnol..

[24] Gu FX (2007). Targeted nanoparticles for cancer therapy. Nano Today.

[25] Steichen SD, Caldorera-Moore M, Peppas NA (2013). A review of current nanoparticle and targeting moieties for the delivery of cancer therapeutics. Eur. J. Pharm. Sci..

[26] Wu X, Chen J, Wu M, Zhao JXJ (2015). Aptamers: active targeting ligands for cancer diagnosis and therapy. Theranostics.

[27] Tan Y (2020). Precisely regulated luminescent gold nanoparticles for identification of cancer metastases. ACS Nano.

[28] Cabral H, Kinoh H, Kataoka K (2020). Tumor-targeted nanomedicine for immunotherapy. Acc. Chem. Res..

[29] Liu GN (2019). Engineering biomimetic platesomes for ph-responsive drug delivery and enhanced antitumor activity. Adv. Mater..

[30] Chiappini C (2015). Biodegradable silicon nanoneedles delivering nucleic acids intracellularly induce localized in vivo neovascularization. Nat. Mater..

[31] Wu D, Wang S, Yu GC, Chen XY (2021). Cell death mediated by the pyroptosis pathway with the aid of nanotechnology: Prospects for cancer therapy. Angew. Chem. Int. Ed..

[32] Ovais M (2020). Designing stimuli-responsive upconversion nanoparticles that exploit the tumor microenvironment. Adv. Mater..

[33] Li L, Yang Z, Chen XY (2020). Recent advances in stimuli-responsive platforms for cancer immunotherapy. Acc. Chem. Res..

[34] Zhou L, Wang H, Li YP (2018). Stimuli-responsive nanomedicines for overcoming cancer multidrug resistance. Theranostics.

[35] Vormittag P, Gunn R, Ghorashian S, Veraitch FS (2018). A guide to manufacturing CAR T cell therapies. Curr. Opin. Biotechnol..

[36] Rosenberg SA, Restifo NP (2015). Adoptive cell transfer as personalized immunotherapy for human cancer. Science.

[37] Shih RM, Chen YY (2022). Engineering principles for synthetic biology circuits in cancer immunotherapy. Cancer Immunol. Res..

[38] Tan X, Letendre JH, Collins JJ, Wong WW (2021). Synthetic biology in the clinic: engineering vaccines, diagnostics, and therapeutics. Cell.

[39] McNerney MP, Doiron KE, Ng TL, Chang TZ, Silver PA (2021). Theranostic cells: emerging clinical applications of synthetic biology. Nat. Rev. Genet..

[40] Cubillos-Ruiz A (2021). Engineering living therapeutics with synthetic biology. Nat. Rev. Drug Discov..

[41] Guo M, Huang K, Xu W (2020). Third generation whole-cell sensing systems: synthetic biology inside, nanomaterial outside. Trends Biotechnol..

[42] Wagner HJ (2019). Synthetic biology-inspired design of signal-amplifying materials systems. Mater. Today.

[43] Guo JS (2014). Click chemistry plays a dual role in biodegradable polymer design. Adv. Mater..

[44] Sletten EM, Bertozzi CR (2011). From mechanism to mouse: a tale of two bioorthogonal reactions. Acc. Chem. Res..

[45] Saxon E, Bertozzi CR (2000). Cell surface engineering by a modified Staudinger reaction. Science.

[46] Lin FL, Hoyt HM, van Halbeek H, Bergman RG, Bertozzi CR (2005). Mechanistic investigation of the Staudinger ligation. J. Am. Chem. Soc..

[47] van Berkel SS, van Eldijk MB, van Hest JCM (2011). Staudinger ligation as a method for bioconjugation. Angew. Chem. Int. Ed..

[48] Bednarek C, Wehl I, Jung N, Schepers U, Brase S (2020). The staudinger ligation. Chem. Rev..

[49] Tornøe CW, Christensen C, Meldal M (2002). Peptidotriazoles on solid phase: 1,2,3-triazoles by regiospecific copper(I)-catalyzed 1,3-dipolar cycloadditions of terminal alkynes to azides. J. Org. Chem..

[50] Rostovtsev VV, Green LG, Fokin VV, Sharpless KB (2002). A stepwise huisgen cycloaddition process: Copper(I)-catalyzed regioselective “ligation” of azides and terminal alkynes. Angew. Chem. Int. Ed..

[51] Kennedy DC (2011). Cellular consequences of copper complexes used to catalyze bioorthogonal click reactions. J. Am. Chem. Soc..

[52] Manova R, van Beek TA, Zuilhof H (2011). Surface functionalization by strain-promoted alkyne-azide click reactions. Angew. Chem. Int. Ed..

[53] Bevilacqua V (2014). Copper-chelating azides for efficient click conjugation reactions in complex media. Angew. Chem. Int. Ed..

[54] Agard NJ, Prescher JA, Bertozzi CR (2005). A strain-promoted 3+2 azide-alkyne cycloaddition for covalent modification of biomolecules in living systems. J. Am. Chem. Soc..

[55] Baskin JM (2007). Copper-free click chemistry for dynamic in vivo imaging. Proc. Natl Acad. Sci. USA.

[56] Sletten EM, Bertozzi CR (2008). A hydrophilic azacyclooctyne for Cu-free click chemistry. Org. Lett..

[57] Blackman ML, Royzen M, Fox JM (2008). Tetrazine ligation: fast bioconjugation based on inverse-electron-demand Diels-Alder reactivity. J. Am. Chem. Soc..

[58] Devaraj NK, Weissleder R, Hilderbrand SA (2008). Tetrazine-based cycloadditions: application to pretargeted live cell imaging. Bioconjugate Chem..

[59] Oliveira BL, Guo Z, Bernardes GJL (2017). Inverse electron demand Diels-Alder reactions in chemical biology. Chem. Soc. Rev..

[60] Row RD, Prescher JA (2018). Constructing new bioorthogonal reagents and reactions. Acc. Chem. Res..

[61] Latocheski E (2020). Mechanistic insights into transition metal-mediated bioorthogonal uncaging reactions. Chem. Soc. Rev..

[62] Lo KKW (2020). Molecular design of bioorthogonal probes and imaging reagents derived from photofunctional transition metal complexes. Acc. Chem. Res..

[63] Parker CG, Pratt MR (2020). Click chemistry in proteomic investigations. Cell.

[64] Kiick KL, Saxon E, Tirrell DA, Bertozzi CR (2002). Incorporation of azides into recombinant proteins for chemoselective modification by the Staudinger ligation. Proc. Natl Acad. Sci. USA.

[65] Agard NJ, Bertozzi CR (2009). Chemical approaches to perturb, profile, and perceive glycans. Acc. Chem. Res..

[66] Du J (2009). Metabolic glycoengineering: sialic acid and beyond. Glycobiology.

[67] Spate AK (2014). Rapid labeling of metabolically engineered cell-surface glycoconjugates with a carbamate-linked cyclopropene reporter. Bioconjugate Chem..

[68] Ricks TJ (2019). Labeling of phosphatidylinositol lipid products in cells through metabolic engineering by using a clickable myo-inositol probe. Chembiochem.

[69] Rahim MK, Kota R, Lee S, Haun JB (2013). Bioorthogonal chemistries for nanomaterial conjugation and targeting. Nanotechnol. Rev..

[70] Meghani NM, Amin HH, Leel BJ (2017). Mechanistic applications of click chemistry for pharmaceutical drug discovery and drug delivery. Drug Discov. Today.

[71] Zhang X, Zhang Y (2013). Applications of azide-based bioorthogonal click chemistry in glycobiology. Molecules.

[72] Agatemor C (2019). Exploiting metabolic glycoengineering to advance healthcare. Nat. Rev. Chem..

[73] Wang H (2019). In vivo cancer targeting via glycopolyester nanoparticle mediated metabolic cell labeling followed by click reaction. Biomaterials.

[74] Yoon HY, Koo H, Kim K, Kwon IC (2017). Molecular imaging based on metabolic glycoengineering and bioorthogonal click chemistry. Biomaterials.

[75] Tomas RMF, Gibson MI (2020). 100th anniversary of macromolecular science viewpoint: re-engineering cellular interfaces with synthetic macromolecules using metabolic glycan labeling. ACS Macro Lett..

[76] Huang LL, Nie W, Zhang J, Xie HY (2020). Cell-membrane-based biomimetic systems with bioorthogonal functionalities. Acc. Chem. Res..

[77] Taiariol L, Chaix C, Farre C, Moreau E (2022). Click and bioorthogonal chemistry: the future of active targeting of nanoparticles for nanomedicines?. Chem. Rev..

[78] Wang H, Mooney DJ (2020). Metabolic glycan labelling for cancer-targeted therapy. Nat. Chem..

[79] Prescher JA, Bertozzi CR (2005). Chemistry in living systems. Nat. Chem. Biol..

[80] Hao ZY, Hong SL, Chen X, Chen PR (2011). Introducing bioorthogonal functionalities into proteins in living cells. Acc. Chem. Res..

[81] Borrmann A, van Hest JCM (2014). Bioorthogonal chemistry in living organisms. Chem. Sci..

[82] Han Y (2019). T cell membrane mimicking nanoparticles with bioorthogonal targeting and immune recognition for enhanced photothermal therapy. Adv. Sci..

[83] Zhang F (2018). Construction of a biomimetic magnetosome and its application as a sirna carrier for high-performance anticancer therapy. Adv. Funct. Mater..

[84] Sun YT (2018). Mechanistic investigation and multiplexing of liposome-assisted metabolic glycan labeling. J. Am. Chem. Soc..

[85] Hu F (2018). A Light-up probe with aggregation-induced emission for real-time bio-orthogonal tumor labeling and image-guided photodynamic therapy. Angew. Chem. Int. Ed..

[86] Yoon HY (2017). Artificial chemical reporter targeting strategy using bioorthogonal click reaction for improving active-targeting efficiency of tumor. Mol. Pharm..

[87] Shim MK (2016). Cathepsin B-specific metabolic precursor for in vivo tumor-specific fluorescence imaging. Angew. Chem. Int. Ed..

[88] Neves AA (2016). Imaging glycosylation in vivo by metabolic labeling and magnetic resonance imaging. Angew. Chem. Int. Ed..

[89] Xie R (2014). Targeted imaging and proteomic analysis of tumor-associated glycans in living animals. Angew. Chem. Int. Ed..

[90] Lee SKH (2014). Chemical tumor-targeting of nanoparticles based on metabolic glycoengineering and click chemistry. ACS Nano.

[91] Xie R, Hong SL, Feng LS, Rong J, Chen X (2012). Cell-selective metabolic glycan labeling based on ligand-targeted liposomes. J. Am. Chem. Soc..

[92] Wang H (2016). In vivo targeting of metabolically labeled cancers with ultra-small silica nanoconjugates. Theranostics.

[93] Chang PV (2009). Metabolic labeling of sialic acids in living animals with alkynyl sugars. Angew. Chem. Int. Ed..

[94] Hsu TL (2007). Alkynyl sugar analogs for the labeling and visualization of glycoconjugates in cells. Proc Natl Acad. Sci. USA.

[95] Wainman YA (2013). Dual-sugar imaging using isonitrile and azido-based click chemistries. Org. Biomol. Chem..

[96] Stairs S (2013). Metabolic glycan imaging by isonitrile-tetrazine click chemistry. Chembiochem.

[97] Lee SY (2016). Non-invasive stem cell tracking in hindlimb ischemia animal model using bio-orthogonal copper-free click chemistry. Biochem. Biophys. Res. Commun..

[98] Wang H (2017). Selective in vivo metabolic cell-labeling-mediated cancer targeting. Nat. Chem. Biol..

[99] Yarema KJ, Mahal LK, Bruehl RE, Rodriguez EC, Bertozzi CR (1998). Metabolic delivery of ketone groups to sialic acid residues-Application to cell surface glycoform engineering. J. Biol. Chem..

[100] Takayama Y, Kusamori K, Nishikawa M (2019). Click chemistry as a tool for cell engineering and drug delivery. Molecules.

[101] Agarwal P, Beahm BJ, Shieh P, Bertozzi CR (2015). Systemic fluorescence imaging of zebrafish glycans with bioorthogonal chemistry. Angew. Chem. Int. Ed..

[102] Lang K, Chin JW (2014). Bioorthogonal reactions for labeling proteins. ACS Chem. Biol..

[103] Dieterich DC, Link AJ, Graumann J, Tirrell DA, Schuman EM (2006). Selective identification of newly synthesized proteins in mammalian cells using bioorthogonal noncanonical amino acid tagging (BONCAT). Proc. Natl Acad. Sci. USA.

[104] Link AJ, Mock ML, Tirrell DA (2003). Non-canonical amino acids in protein engineering. Curr. Opin. Biotechnol..

[105] van Hest JCM, Kiick KL, Tirrell DA (2000). Efficient incorporation of unsaturated methionine analogues into proteins in vivo. J. Am. Chem. Soc..

[106] Huang LL (2013). Enveloped virus labeling via both intrinsic biosynthesis and metabolic incorporation of phospholipids in host cells. Anal. Chem..

[107] Jao CY, Roth M, Welti R, Salic A (2009). Metabolic labeling and direct imaging of choline phospholipids in vivo. Proc. Natl Acad. Sci. USA.

[108] Hao MX (2020). Combination of metabolic intervention and T cell therapy enhances solid tumor immunotherapy. Sci. Transl. Med..

[109] Qin H (2021). Development of a cancer vaccine using in vivo click-chemistry-mediated active lymph node accumulation for improved immunotherapy. Adv. Mater..

[110] Xiao P (2021). Engineering nanoscale artificial antigen-presenting cells by metabolic dendritic cell labeling to potentiate cancer immunotherapy. Nano Lett..

[111] Wang H (2020). Metabolic labeling and targeted modulation of dendritic cells. Nat. Mater..

[112] Koo H (2012). Bioorthogonal copper-free click chemistry in vivo for tumor-targeted delivery of nanoparticles. Angew. Chem. Int. Ed..

[113] Du L, Qin H, Ma T, Zhang T, Xing D (2017). In vivo imaging-guided photothermal/photoacoustic synergistic therapy with bioorthogonal metabolic glycoengineering-activated tumor targeting nanoparticles. ACS Nano.

[114] Wang H (2016). Targeted ultrasound-assisted cancer-selective chemical labeling and subsequent cancer imaging using click chemistry. Angew. Chem. Int. Ed..

[115] Lee S (2017). Nano-sized metabolic precursors for heterogeneous tumor-targeting strategy using bioorthogonal click chemistry in vivo. Biomaterials.

[116] Li S (2018). Biomarker-based metabolic labeling for redirected and enhanced immune response. ACS Chem. Biol..

[117] Chang PV, Dube DH, Sletten EM, Bertozzi CR (2010). A strategy for the selective imaging of glycans using caged metabolic precursors. J. Am. Chem. Soc..

[118] Tra VN, Dube DH (2014). Glycans in pathogenic bacteria-potential for targeted covalent therapeutics and imaging agents. Chem. Commun..

[119] Swarts BM (2012). Probing the mycobacterial trehalome with bioorthogonal chemistry. J. Am. Chem. Soc..

[120] Lam H (2009). D-amino acids govern stationary phase cell wall remodeling in bacteria. Science.

[121] Huang BH (2012). Surface labeling of enveloped viruses assisted by host cells. ACS Chem. Biol..

[122] Huang LL (2018). Labeling and single-particle-tracking-based entry mechanism study of vaccinia virus from the tiantan strain. Anal. Chem..

[123] Pan H (2017). In situ bioorthogonal metabolic labeling for fluorescence imaging of virus infection in vivo. Small.

[124] de Melo-Diogo D, Pais-Silva C, Dias DR, Moreira AF, Correia IJ (2017). Strategies to improve cancer photothermal therapy mediated by nanomaterials. Adv. Healthc. Mater..

[125] Bazak R, Houri M, El Achy S, Kamel S, Refaat T (2015). Cancer active targeting by nanoparticles: a comprehensive review of literature. J. Cancer Res. Clin. Oncol..

[126] Byrne JD, Betancourt T, Brannon-Peppas L (2008). Active targeting schemes for nanoparticle systems in cancer therapeutics. Adv. Drug Deliv. Rev..

[127] Saei AA (2017). Nanoparticle surface functionality dictates cellular and systemic toxicity. Chem. Mater..

[128] Pelaz B (2013). Interfacing engineered nanoparticles with biological systems: anticipating adverse nanobio interactions. Small.

[129] Qiao J (2020). Bio-orthogonal click-targeting nanocomposites for chemo-photothermal synergistic therapy in breast cancer. Theranostics.

[130] Viola-Villegas NT (2014). Understanding the pharmacological properties of a metabolic PET tracer in prostate cancer. Proc. Natl Acad. Sci. USA.

[131] Lu GH (2018). Amplifying nanoparticle targeting performance to tumor via Diels-Alder cycloaddition. Adv. Funct. Mater..

[132] Sosunov EA (2013). pH (low) insertion peptide (pHLIP) targets ischemic myocardium. Proc. Natl Acad. Sci. USA.

[133] Lee SH (2019). Deep tumor penetration of drug-loaded nanoparticles by click reaction-assisted immune cell targeting strategy. J. Am. Chem. Soc..

[134] Sun QH, Zhou ZX, Qiu NS, Shen YQ (2017). Rational design of cancer nanomedicine: nanoproperty integration and synchronization. Adv. Mater..

[135] Tong, R. & Kohane, D. S. In *Annual review of pharmacology and toxicology*, *Vol. 56* (ed Insel, P. A.) 41–57 (Annual Reviews, 2016). 10.1146/annurev-pharmtox-010715-103456 26514197

[136] Tu YL (2020). Intercellular delivery of bioorthogonal chemical receptors for enhanced tumor targeting and penetration. Biomaterials.

[137] Sindhwani S (2020). The entry of nanoparticles into solid tumours. Nat. Mater..

[138] Farokhzad OC, Langer R (2009). Impact of nanotechnology on drug delivery. ACS Nano.

[139] Wang KW (2022). Tumor-acidity and bioorthogonal chemistry-mediated on-site size transformation clustered nanosystem to overcome hypoxic resistance and enhance chemoimmunotherapy. ACS Nano.

[140] Jiang M, Liu Y, Dong Y, Wang K, Yuan Y (2022). Bioorthogonal chemistry and illumination controlled programmed size-changeable nanomedicine for synergistic photodynamic and hypoxia-activated therapy. Biomaterials.

[141] Gao J (2022). Engineered bioorthogonal POLY-PROTAC nanoparticles for tumour-specific protein degradation and precise cancer therapy. Nat. Commun..

[142] Cao Z (2022). Bioorthogonal in situ assembly of nanomedicines as drug depots for extracellular drug delivery. Nat. Commun..

[143] Tan WH, Donovan MJ, Jiang JH (2013). Aptamers from cell-based selection for bioanalytical applications. Chem. Rev..

[144] Yang Y (2021). Aptamer-based logic computing reaction on living cells to enable non-antibody immune checkpoint blockade therapy. J. Am. Chem. Soc..

[145] Ge XG (2019). Photoacoustic imaging and photothermal therapy in the second near-infrared window. N. J. Chem..

[146] Li BH, Lu LF, Zhao MY, Lei ZH, Zhang F (2018). An efficient 1064 nm NIR-II excitation fluorescent molecular dye for deep-tissue high-resolution dynamic bioimaging. Angew. Chem. Int. Ed..

[147] Jiang YY, Pu KY (2018). Multimodal biophotonics of semiconducting polymer nanoparticles. Acc. Chem. Res..

[148] Zhang WS (2020). Bioorthogonal-targeted 1064 nm excitation theranostic nanoplatform for precise NIR-IIa fluorescence imaging guided efficient NIR-II photothermal therapy. Biomaterials.

[149] Jentsch TJ, Hubner CA, Fuhrmann JC (2004). Ion channels: function unravelled by dysfunction. Nat. Cell Biol..

[150] Lin JY, Knutsen PM, Muller A, Kleinfeld D, Tsien RY (2013). ReaChR: a red-shifted variant of channelrhodopsin enables deep transcranial optogenetic excitation. Nat. Neurosci..

[151] Kobayashi H, Ogawa M, Alford R, Choyke PL, Urano Y (2010). New strategies for fluorescent probe design in medical diagnostic imaging. Chem. Rev..

[152] Ai XZ (2017). Remote regulation of membrane channel activity by site-specific localization of lanthanide-doped upconversion nanocrystals. Angew. Chem. Int. Ed..

[153] Song L (2018). Low-dose x-ray activation of w(VI)-doped persistent luminescence nanoparticles for deep-tissue photodynamic therapy. Adv. Funct. Mater..

[154] Ni KY (2018). Nanoscale metal-organic frameworks for mitochondria-targeted radiotherapy-radiodynamic therapy. Nat. Commun..

[155] Lu KD (2018). Low-dose X-ray radiotherapy-radiodynamic therapy via nanoscale metal-organic frameworks enhances checkpoint blockade immunotherapy. Nat. Biomed. Eng..

[156] Hu F, Xu SD, Liu B (2018). Photosensitizers with aggregation-induced emission: materials and biomedical applications. Adv. Mater..

[157] Mei J, Leung NLC, Kwok RTK, Lam JWY, Tang BZ (2015). Aggregation-induced emission: together we shine, united we soar!. Chem. Rev..

[158] Liu JJ (2021). Bioorthogonal coordination polymer nanoparticles with aggregation-induced emission for deep tumor-penetrating radio- and radiodynamic therapy. Adv. Mater..

[159] Larson SM, Carrasquillo JA, Cheung NKV, Press OW (2015). Radioimmunotherapy of human tumours. Nat. Rev. Cancer.

[160] Patra M, Zarschler K, Pietzsch HJ, Stephan H, Gasser G (2016). New insights into the pretargeting approach to image and treat tumours. Chem. Soc. Rev..

[161] Shah MA (2017). Metal-free cycloaddition chemistry driven pretargeted radioimmunotherapy using alpha-particle radiation. Bioconjugate Chem..

[162] van der Burg SH, Arens R, Ossendorp F, van Hall T, Melief AJM (2016). Vaccines for established cancer: overcoming the challenges posed by immune evasion. Nat. Rev. Cancer.

[163] Branca MA (2016). Rekindling cancer vaccines. Nat. Biotechnol..

[164] Li HY, Li YH, Jiao J, Hu HM (2011). Alpha-alumina nanoparticles induce efficient autophagy-dependent cross-presentation and potent antitumour response. Nat. Nanotechnol..

[165] Van Herck S (2018). Lymph-node-targeted immune activation by engineered block copolymer amphiphiles-TLR7/8 agonist conjugates. J. Am. Chem. Soc..

[166] Nuhn L (2018). Nanoparticle-conjugate TLR7/8 agonist localized immunotherapy provokes safe antitumoral responses. Adv. Mater..

[167] Zhu GZ, Zhang FW, Ni QQ, Niu G, Chen XY (2017). Efficient nanovaccine delivery in cancer immunotherapy. ACS Nano.

[168] Liu HP (2014). Structure-based programming of lymph-node targeting in molecular vaccines. Nature.

[169] Takeda A (2019). Single-cell survey of human lymphatics unveils marked endothelial cell heterogeneity and mechanisms of homing for neutrophils. Immunity.

[170] Hu XX, Zhong L, Zhang X, Gao YM, Liu BZ (2014). NLS-RAR alpha promotes proliferation and inhibits differentiation in HL-60 cells. Int. J. Med. Sci..

[171] Hinrichs CS, Rosenberg SA (2014). Exploiting the curative potential of adoptive T-cell therapy for cancer. Immunol. Rev..

[172] Guo B (2016). CD138-directed adoptive immunotherapy of chimeric antigen receptor (CAR)-modified T cells for multiple myeloma. J. Cell. Immunother..

[173] Pan H (2019). Glycometabolic bioorthogonal chemistry-guided viral transduction for robust human t cell engineering. Adv. Funct. Mater..

[174] van Oosten M (2013). Real-time in vivo imaging of invasive- and biomaterial-associated bacterial infections using fluorescently labelled vancomycin. Nat. Commun..

[175] Chen J, Andler SM, Goddard JM, Nugen SR, Rotello VM (2017). Integrating recognition elements with nanomaterials for bacteria sensing. Chem. Soc. Rev..

[176] Mao D (2018). Metal-organic-framework-assisted in vivo bacterial metabolic labeling and precise antibacterial therapy. Adv. Mater..

[177] Hou S (2021). Metabolic labeling mediated targeting and thermal killing of gram-positive bacteria by self-reporting janus magnetic nanoparticles. Small.

[178] Liang J, Tang B, Liu B (2015). Specific light-up bioprobes based on AIEgen conjugates. Chem. Soc. Rev..

[179] Wu M (2020). Bio-orthogonal AlEgen for specific discrimination and elimination of bacterial pathogens via metabolic engineering. Chem. Mater..

[180] Hooper LV, Littman DR, Macpherson AJ (2012). Interactions between the microbiota and the immune system. Science.

[181] Juul FE (2018). Fecal microbiota transplantation for primary clostridium difficile infection. N. Engl. J. Med..

[182] Song WF (2022). In situ bioorthogonal conjugation of delivered bacteria with gut inhabitants for enhancing probiotics colonization. ACS Cent. Sci..

[183] Jia H-R (2022). Recent advances of cell surface modification based on aptamers. Mater. Today Nano.

[184] Wang X (2019). Copper-triggered bioorthogonal cleavage reactions for reversible protein and cell surface modifications. J. Am. Chem. Soc..

[185] Amani H (2019). Controlling cell behavior through the design of biomaterial surfaces: a focus on surface modification techniques. Adv. Mater. Interfaces.

[186] Pulsipher A, Griffin ME, Stone SE, Brown JM, Hsieh-Wilson LC (2014). Directing neuronal signaling through cell-surface glycan engineering. J. Am. Chem. Soc..

[187] Paszek MJ (2014). The cancer glycocalyx mechanically primes integrin-mediated growth and survival. Nature.

[188] Liu J, Liew SS, Wang J, Pu KY (2022). Bioinspired and biomimetic delivery platforms for cancer vaccines. Adv. Mater..

[189] Lai X (2022). Light-triggered efficient sequential drug delivery of biomimetic nanosystem for multimodal chemo-, antiangiogenic, and anti-MDSC therapy in melanoma. Adv. Mater..

[190] Rahamim V, Azagury A (2021). Bioengineered biomimetic and bioinspired noninvasive drug delivery systems. Adv. Funct. Mater..

[191] Gong X (2019). Emerging approaches of cell-based nanosystems to target cancer metastasis. Adv. Funct. Mater..

[192] Liu LZ (2021). Cell membrane coating integrity affects the internalization mechanism of biomimetic nanoparticles. Nat. Commun..

[193] Baruch EN, Berg AL, Besser MJ, Schachter J, Markel G (2017). Adoptive T cell therapy: an overview of obstacles and opportunities. Cancer.

[194] Li W (2018). Bio-orthogonal t cell targeting strategy for robustly enhancing cytotoxicity against tumor cells. Small.

[195] Pan H (2021). Click CAR-T cell engineering for robustly boosting cell immunotherapy in blood and subcutaneous xenograft tumor. Bioact. Mater..

[196] Xiong K (2016). Biomimetic immuno-magnetosomes for high-performance enrichment of circulating tumor cells. Adv. Mater..

[197] Fierer JO, Veggiani G, Howarth M (2014). SpyLigase peptide-peptide ligation polymerizes affibodies to enhance magnetic cancer cell capture. Proc. Natl Acad. Sci. USA.

[198] Zhang QZ (2020). Cellular nanosponges inhibit SARS-CoV-2 infectivity. Nano Lett..

[199] Clausen TM (2020). SARS-CoV-2 infection depends on cellular heparan sulfate and ACE2. Cell.

[200] Tandon R (2021). Effective inhibition of SARS-CoV-2 entry by heparin and enoxaparin derivatives. J. Virol..

[201] Ai XZ (2021). Surface glycan modification of cellular nanosponges to promote SARS-CoV-2 inhibition. J. Am. Chem. Soc..

[202] Waldman AD, Fritz JM, Lenardo MJ (2020). A guide to cancer immunotherapy: from T cell basic science to clinical practice. Nat. Rev. Immunol..

[203] Irvine DJ, Swartz MA, Szeto GL (2013). Engineering synthetic vaccines using cues from natural immunity. Nat. Mater..

[204] Dudziak D (2007). Differential antigen processing by dendritic cell subsets in vivo. Science.

[205] Li F (2019). Engineering magnetosomes for high-performance cancer vaccination. ACS Cent. Sci..

[206] Tang ZM, Liu YY, He MY, Bu WB (2019). Chemodynamic therapy: tumour microenvironment-mediated fenton and fenton-like reactions. Angew. Chem. Int. Ed..

[207] Lisse D (2017). Engineered ferritin for magnetogenetic manipulation of proteins and organelles inside living cells. Adv. Mater..

[208] Zhang F (2019). Engineering magnetosomes for ferroptosis/immunomodulation synergism in cancer. ACS Nano.

[209] Wu PP, Zhang B, Ocansey DKW, Wenrong, Qian H (2021). Extracellular vesicles: a bright star of nanomedicine. Biomaterials.

[210] Liang YJ, Duan L, Lu JP, Xia J (2021). Engineering exosomes for targeted drug delivery. Theranostics.

[211] Nie WD (2020). Responsive exosome nano-bioconjugates for synergistic cancer therapy. Angew. Chem. Int. Ed..

[212] Zhang L (2022). Nanoenzyme engineered neutrophil-derived exosomes attenuate joint injury in advanced rheumatoid arthritis via regulating inflammatory environment. Bioact. Mater..

[213] Sun L (2022). Nanoengineered neutrophils as a cellular sonosensitizer for visual sonodynamic therapy of malignant tumors. Adv. Mater..

[214] Nemeth T, Sperandio M, Mocsai A (2020). Neutrophils as emerging therapeutic targets. Nat. Rev. Drug Discov..

[215] Zhang L (2019). Transforming weakness into strength: photothermal-therapy-induced inflammation enhanced cytopharmaceutical chemotherapy as a combination anticancer treatment. Adv. Mater..

[216] Chu D, Dong X, Shi X, Zhang C, Wang Z (2018). Neutrophil-based drug delivery systems. Adv. Mater..

[217] Xue J (2017). Neutrophil-mediated anticancer drug delivery for suppression of postoperative malignant glioma recurrence. Nat. Nanotechnol..

[218] Weyand CM, Goronzy JJ (2017). Immunometabolism in early and late stages of rheumatoid arthritis. Nat. Rev. Rheumatol..

[219] Prakash S, Kumbhojkar N, Clegg JR, Mitragotri S (2021). Cell-bound nanoparticles for tissue targeting and immunotherapy: Engineering of the particle-membrane interface. Curr. Opin. Colloid Interface Sci..

[220] Zhao ZM (2020). Engineering of living cells with polyphenol-functionalized biologically active nanocomplexes. Adv. Mater..

[221] Shields CW (2020). Cellular backpacks for macrophage immunotherapy. Sci. Adv..

[222] Polak R (2015). Liposome-loaded cell backpacks. Adv. Healthc. Mater..

[223] June CH, O’Connor RS, Kawalekar OU, Ghassemi S, Milone MC (2018). CAR T cell immunotherapy for human cancer. Science.

[224] Overchuk M, Zheng G (2018). Overcoming obstacles in the tumor microenvironment: recent advancements in nanoparticle delivery for cancer theranostics. Biomaterials.

[225] Chen Z (2021). Nanoengineered CAR-T biohybrids for solid tumor immunotherapy with microenvironment photothermal-remodeling strategy. Small.

[226] Luo Y (2022). IL-12 nanochaperone-engineered CAR T cell for robust tumor-immunotherapy. Biomaterials.

[227] Yang W (2016). Potentiating the antitumour response of CD8(+) T cells by modulating cholesterol metabolism. Nature.

[228] Lee S (2017). In vivo stem cell tracking with imageable nanoparticles that bind bioorthogonal chemical receptors on the stem cell surface. Biomaterials.

[229] Layek B, Sadhukha T, Prabha S (2016). Glycoengineered mesenchymal stem cells as an enabling platform for two-step targeting of solid tumors. Biomaterials.

[230] Mimpen M, Smolders J, Hupperts R, Damoiseaux J (2020). Natural killer cells in multiple sclerosis: a review. Immunol. Lett..

[231] Lamb MG, Rangarajan HG, Tullius BP, Lee DA (2021). Natural killer cell therapy for hematologic malignancies: successes, challenges, and the future. Stem Cell Res. Ther..

[232] Meng D (2022). In situ activated NK cell as bio-orthogonal targeted live-cell nanocarrier augmented solid tumor immunotherapy. Adv. Funct. Mater..

[233] Matosevic S (2018). Viral and nonviral engineering of natural killer cells as emerging adoptive cancer immunotherapies. J. Immunol. Res..

[234] Wang X (2020). Glycoengineering of natural killer cells with CD22 ligands for enhanced anticancer immunotherapy. ACS Cent. Sci..

[235] Zhao Y (2022). Bioorthogonal equipping CAR-T cells with hyaluronidase and checkpoint blocking antibody for enhanced solid tumor immunotherapy. ACS Cent. Sci..

[236] Au KM, Medik Y, Ke Q, Tisch R, Wang AZ (2021). Immune checkpoint-bioengineered beta cell vaccine reverses early-onset type 1 diabetes. Adv. Mater..

[237] Ben Nasr M (2017). PD-L1 genetic overexpression or pharmacological restoration in hematopoietic stem and progenitor cells reverses autoimmune diabetes. Sci. Transl. Med..

[238] Wen X (2008). Transplantation of NIT-1 cells expressing pD-L1 for treatment of streptozotocin-induced diabetes. Transplantation.

[239] Dahlen E, Hedlund G, Dawe K (2000). Low CD86 expression in the nonobese diabetic mouse results in the impairment of both T cell activation and CTLA-4 up-regulation. J. Immunol..

[240] Sato K (2018). Photoinduced ligand release from a silicon phthalocyanine dye conjugated with monoclonal antibodies: a mechanism of cancer cell cytotoxicity after near-infrared photoimmunotherapy. ACS Cent. Sci..

[241] Komatsu T (2020). Antibody clicking as a strategy to modify antibody functionalities on the surface of targeted cells. J. Am. Chem. Soc..

[242] Hu Q (2018). Conjugation of haematopoietic stem cells and platelets decorated with anti-PD-1 antibodies augments anti-leukaemia efficacy. Nat. Biomed. Eng..

[243] Li Z (2021). Chemically and biologically engineered bacteria-based delivery systems for emerging diagnosis and advanced therapy. Adv. Mater..

[244] Pan P (2019). Bio-orthogonal bacterial reactor for remission of heavy metal poisoning and ROS elimination. Adv. Sci..

[245] Wang HJ (2019). Bacteria-induced aggregation of bioorthogonal gold nanoparticles for SERS imaging and enhanced photothermal ablation of Gram-positive bacteria. J. Mater. Chem. B.

[246] Foglizzo V, Marchio S (2021). Bacteriophages as therapeutic and diagnostic vehicles in cancer. Pharmaceuticals.

[247] Sunderland KS, Yang MY, Mao CB (2017). Phage-enabled nanomedicine: from probes to therapeutics in precision medicine. Angew. Chem. Int. Ed..

[248] Ju ZG, Sun W (2017). Drug delivery vectors based on filamentous bacteriophages and phage-mimetic nanoparticles. Drug Deliv..

[249] Zheng DW (2019). Phage-guided modulation of the gut microbiota of mouse models of colorectal cancer augments their responses to chemotherapy. Nat. Biomed. Eng..

[250] Xue K (2019). Hydrogels as emerging materials for translational biomedicine. Adv. Therapeu.

[251] Annabi N (2014). 25th anniversary article: rational design and applications of hydrogels in regenerative medicine. Adv. Mater..

[252] Yu L, Ding JD (2008). Injectable hydrogels as unique biomedical materials. Chem. Soc. Rev..

[253] Shirbin SJ, Karimi F, Chan NJA, Heath DE, Qiao GG (2016). Macroporous hydrogels composed entirely of synthetic polypeptides: biocompatible and enzyme biodegradable 3D cellular scaffolds. Biomacromolecules.

[254] Chen F, Le P, Fernandes-Cunha GM, Heilshorn SC, Myung D (2020). Bio-orthogonally crosslinked hyaluronate-collagen hydrogel for suture-free corneal defect repair. Biomaterials.

[255] Rong Y, Zhang Z, He C, Chen X (2020). Bioactive polypeptide hydrogels modified with RGD and N-cadherin mimetic peptide promote chondrogenic differentiation of bone marrow mesenchymal stem cells. Sci. China Chem..

[256] Kim T, Suh J, Kim WJ (2021). Polymeric aggregate-embodied hybrid nitric-oxide-scavenging and sequential drug-releasing hydrogel for combinatorial treatment of rheumatoid arthritis. Adv. Mater..

[257] Xing Y (2022). Engineering pro-angiogenic biomaterials via chemoselective extracellular vesicle immobilization. Biomaterials.

[258] Wratil PR, Horstkorte R, Reutter W (2016). Metabolic glycoengineering with N-Acyl side chain modified mannosamines. Angew. Chem. Int. Ed..

[259] Hou XS, Ke CF, Stoddart JF (2016). Cooperative capture synthesis: yet another playground for copper-free click chemistry. Chem. Soc. Rev..

[260] Knall AC, Slugovc C (2013). Inverse electron demand Diels-Alder (iEDDA)-initiated conjugation: a (high) potential click chemistry scheme. Chem. Soc. Rev..

[261] Deng ZY, Liu SY (2020). Controlled drug delivery with nanoassemblies of redox-responsive prodrug and polyprodrug amphiphiles. J. Control. Release.

[262] Zhang Y (2017). Strategies for improving the payload of small molecular drugs in polymeric micelles. J. Control. Release.

[263] Wen J (2017). Diverse gatekeepers for mesoporous silica nanoparticle based drug delivery systems. Chem. Soc. Rev..

[264] Zhou M (2016). Photo-click construction of a targetable and activatable two-photon probe imaging protease in apoptosis. Chem. Commun..

[265] Huang K (2019). Small, traceable, endosome-disrupting, and bioresponsive click nanogels fabricated via microfluidics for CD44-targeted cytoplasmic delivery of therapeutic proteins. ACS Appl. Mater. Interfaces.

[266] Tang L (2018). Enhancing T cell therapy through TCR-signaling-responsive nanoparticle drug delivery. Nat. Biotechnol..

[267] Li J, Chen PR (2016). Development and application of bond cleavage reactions in bioorthogonal chemistry. Nat. Chem. Biol..

[268] Liang T, Chen Z, Li H, Gu Z (2022). Bioorthogonal catalysis for biomedical applications. Trends Chem..

[269] Chen Z (2021). Bioorthogonal catalytic patch. Nat. Nanotechnol..

[270] Tessier R (2019). “Doubly orthogonal” labeling of peptides and proteins. Chem.

[271] Zhang, F. et al. Nanoparticle-modified microrobots for in vivo antibiotic delivery to treat acute bacterial pneumonia. *Nat. Mater*. 10.1038/s41563-022-01360-9 (2022). 10.1038/s41563-022-01360-9 PMC9633541 36138145

[272] Cheng Q (2020). Selective organ targeting (SORT) nanoparticles for tissue-specific mRNA delivery and CRISPR–Cas gene editing. Nat. Nanotechnol..

[273] Ngai WSC (2022). Bioorthogonally activatable base editing for on-demand pyroptosis. J. Am. Chem. Soc..

[274] Zhang QB (2017). Biomimetic magnetosomes as versatile artificial antigen-presenting cells to potentiate T-cell-based anticancer therapy. ACS Nano.

